# Transient brain activity disentangles fMRI resting-state dynamics in terms of spatially and temporally overlapping networks

**DOI:** 10.1038/ncomms8751

**Published:** 2015-07-16

**Authors:** Fikret Işik Karahanoğlu, Dimitri Van De Ville

**Affiliations:** 1 Department of Radiology and Medical Informatics, University of Geneva, Geneva 1211, Switzerland; 2 Institute of Bioengineering, Ecole Polytechnique Fédérale de Lausanne, Lausanne 1015, Switzerland; 3 Department of Psychiatry, University of Geneva, Geneva 1211, Switzerland; 4 Athinoula A. Martinos Center for Biomedical Imaging, Harvard Medical School, Boston, Massachusetts 02129, USA

## Abstract

Dynamics of resting-state functional magnetic resonance imaging (fMRI) provide a new window onto the organizational principles of brain function. Using state-of-the-art signal processing techniques, we extract innovation-driven co-activation patterns (iCAPs) from resting-state fMRI. The iCAPs' maps are spatially overlapping and their sustained-activity signals temporally overlapping. Decomposing resting-state fMRI using iCAPs reveals the rich spatiotemporal structure of functional components that dynamically assemble known resting-state networks. The temporal overlap between iCAPs is substantial; typically, three to four iCAPs occur simultaneously in combinations that are consistent with their behaviour profiles. In contrast to conventional connectivity analysis, which suggests a negative correlation between fluctuations in the default-mode network (DMN) and task-positive networks, we instead find evidence for two DMN-related iCAPs consisting the posterior cingulate cortex that differentially interact with the attention network. These findings demonstrate how the fMRI resting state can be functionally decomposed into spatially and temporally overlapping building blocks using iCAPs.

## Abstract (web summary)

Growing evidence from neuroimaging studies suggests that the brain is dynamically organized into functionally connected networks. Here, the authors develop a new technique for decomposing spontaneous activity into temporally overlapping building blocks that assemble standard functional networks.

## Graphical abstract

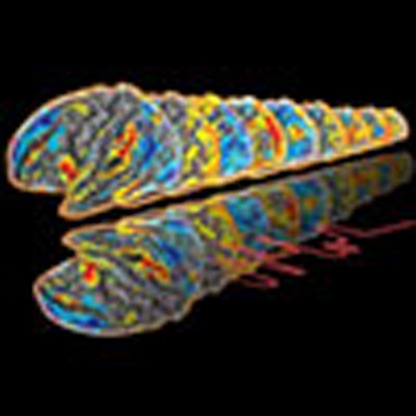

Spontaneous brain activity measured by functional magnetic resonance imaging (fMRI) has provided evidence that the human brain is intrinsically organized into large-scale functional networks^1^. Typically, fluctuations of the blood-oxygenated-level-dependent (BOLD) signals obtained during 5–10 min fMRI resting-state scans have been corroborated to aggregate brain regions with temporally coherent activity. Despite being referred to as resting-state networks (RSNs), these networks are reminiscent of common task-explicit activation patterns related to motor, attention, visual networks^2^,^3^,^4^. In addition, they are reproducible across healthy human individuals and non-human primates, and have been studied not only with fMRI, but also with other imaging modalities including electroencepholography^5^,^6^, electrocorticography^7^ and magnetoencephalography^8^.

Findings based on resting-state fMRI are closely related to underlying data analysis methodologies such as seed correlation analysis^2^, fuzzy clustering^9^, temporal clustering analysis^10^,^11^ or subspace decomposition methods including independent component analysis (ICA)^12^,^13^,^14^, canonical correlation analysis^15^ and agnostic canonical variates analysis^16^. Seed correlation analysis, which builds a connectivity map from correlations with the time course of a preselected seed region, and spatial ICA, which identifies components using a proxy of statistical independence^17^, have been most widely used, but both assume stationary temporal behaviour.

Growing evidence points to the importance of dynamical features of resting-state fMRI data to discover relevant organization of brain function. Different methodologies have been adapted to revisit resting state from this new emerging viewpoint. First, using sliding-window correlation^18^, dynamic functional connectivity can be represented by a limited number of connectivity patterns^19^,^20^,^21^,^22^,^23^. Second, using temporal ICA combined with fast acquisition schemes, temporal functional modes (TFMs)^24^ have been identified. TFMs are spatially overlapping sources optimized to be as independent in time as possible. Third, functional connectivity networks have been classified by latent Dirichlet allocation that allows for spatial overlap^25^. Finally, seed correlation analysis has been extended to extract different co-activation patterns (CAPs) for a predefined seed region^26^,^27^. Inspired by point process analysis^28^, whole-brain activation maps from time points where the seed region's signal exceeds a threshold enter into a temporal clustering step; CAPs are then recovered as the average brain activity maps for the different temporal clusters. These studies provide convincing evidence that conventional RSNs can be decomposed in time by spatially overlapping components, however, both TFMs and CAPs are driven by temporal segregation as one of the underlying assumptions of the analysis. It remains an open question whether dynamics of ongoing activity measured by fMRI can be considered to cycle through temporally segregated states, or whether it is better described by temporally overlapping components that form the RSNs. Identifying the elementary building blocks of ongoing activity and obtaining a better understanding of their temporal organization can then provide new avenues to study their relationship with more temporally precise electrophysiological signals such as EEG and MEG^29^, as well as shed light on changes in neural dynamics in neurodegenerative diseases^30^.

To overcome current limitations in the analysis of resting-state dynamics, we propose to represent spontaneous brain activity by ‘transients' and to explicitly account for temporal blurring by the hemodynamic response function (HRF). Specifically, when the fMRI signal of a region or a network is ‘high', several regions might be co-activated even though their initial onsets are different and thus they could be considered as belonging to different components. Such ambiguity renders it difficult to study the superposed activity of RSNs including their lagging structure^31^. Therefore, we build upon a recent framework for sparsity-pursuing regularization, termed total activation (TA)^32^, to temporally deconvolve fMRI time series. TA makes use of the prior knowledge of the HRF that enables us to use the full-spectrum fMRI signal (that is, without bandpass filtering). By applying TA, we obtain three types of information: (1) activity-related signals that are de-noised fMRI signals, (2) sustained, or block-type, activity-inducing signals that are deconvolved signals, (3) innovation signals that are the derivative of the activity-inducing signals and encode transient brain activity by spikes. We then perform temporal clustering on the whole-brain innovation signals extracted from resting-state fMRI data of 14 healthy volunteers and recover the corresponding spatial patterns, which we refer to as innovation-driven co-activation patterns (iCAPs). We demonstrate that, despite representing short transients in fMRI activity, these iCAPs are robust for both positive and negative transients, and reflect common resting-state patterns. In addition, iCAPs overlap not only spatially, but also temporally when back-projected to their sustained-activity-inducing signals. The total activity of all the iCAPs exceeds three times the duration of the resting-state scan, however, overlapping iCAPs do not co-occur in every possible combination. Clustering iCAPs according to observed combinations reveals a high-level organization of brain function during rest that is consistent with the iCAPs' behaviour profiles^33^. We specifically study iCAPs that relate to well-known resting-state networks such as the default-mode network (DMN) and the attention network. Temporal dynamics of these iCAPs show that activity in the DMN is not always anti-correlated with attention network, as has been assumed on the basis of results from conventional analysis^34^. Instead, segregated subcomponents of the DMN in the posterior cingulate cortex (PCC) differentially interact with the attention network.

## Results

### Transient activity maps are reminiscent of RSNs

**Figure 1:**
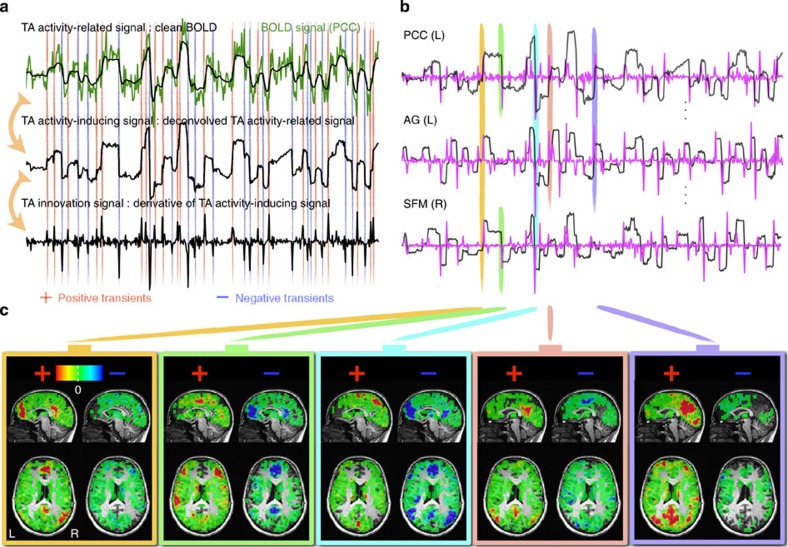
Total activation results. (**a**) Results obtained by applying total activation to a voxel's time course in posterior cingulate cortex (PCC): measured noisy BOLD signal (green) and activity-related signal obtained after TA regularization (top), block-like activity-inducing signal without the hemodynamic blurring (middle) and sparse innovations, derivative of activity-inducing signals, (bottom). (**b**) Activity-inducing signals (black) and innovations (magenta) for PCC (top), angular gyrus (AG) and superior frontal medial cortex (SFM). (**c**) Whole-brain transient activity maps at randomly selected time points indicated by the coloured bars in **b**.

We first illustrate the concept of TA using the data of one representative subject. Specifically, for a voxel in PCC, Fig. 1a depicts the original BOLD signal (top, green) and the de-noised activity-related signal (top, black) obtained by applying TA regularization, the deconvolved activity-inducing signal that is block-type (middle) and has been undone from the effect of the HRF, and the innovation signal (bottom) that is the derivative of activity-inducing signal and thus represents transient activations. The activity-inducing signals (black) and innovation signals (magenta) of voxels in three key regions of the DMN are plotted in Fig. 1b—that is, PCC, angular gyrus (AG) and superior frontal medial cortex.

The innovation signals encode the transients of fMRI BOLD activations and de-activations by positive and negative spikes, respectively. Figure 1c displays the whole-brain transient activity maps at each of the time instances (indicated by orange, green, cyan, pink and violet bars). The first map corresponds to DMN (orange, positive), the second one to inferior frontal and parietal regions (green, positive) and DMN (green, negative), the third indicates DMN (cyan, negative) and superior precuneus and thalamus (cyan, negative), the fourth map reveals posterior DMN (pDMN; pink, positive) and the fifth map highlights mostly pDMN along with cuneus (violet, positive). From this example, it is clear that transient activity maps at time instances of large transients represent meaningful spatial patterns of spontaneous BOLD fluctuations. The various patterns illustrated here, only for three key regions of the DMN, are surprisingly strong in terms of spatial contrast and at the same time reflect the variability over time in how subcomponents of the DMN are activated.

### Clustering transients show spatially overlapping patterns

To systematically study consistency of transient activity maps, we performed clustering of the time instances using the cosine distance between the corresponding whole-brain transient activity maps. First, time instances with significant innovations are determined using a two-step procedure: (1) for every voxel, we mark time instances where the innovation signal exceeds a threshold given by a null hypothesis of stationary data separately driven from each subject (see Supplementary Methods and Supplementary Figs 1 and 2 for details on phase randomization); (2) for each subject, we apply a global thresholding corresponding to at least 500 ‘active' voxels. Second, clustering is then performed on the selected, but non-thresholded transient activity maps, leading to total number of included time instances of 28% (1,521 positive transients, 1,477 negative transients and total 2,998 (56%) out of 5,280 scans). For each temporal cluster, we can then compute a representative iCAP across all subjects.

**Figure 2:**
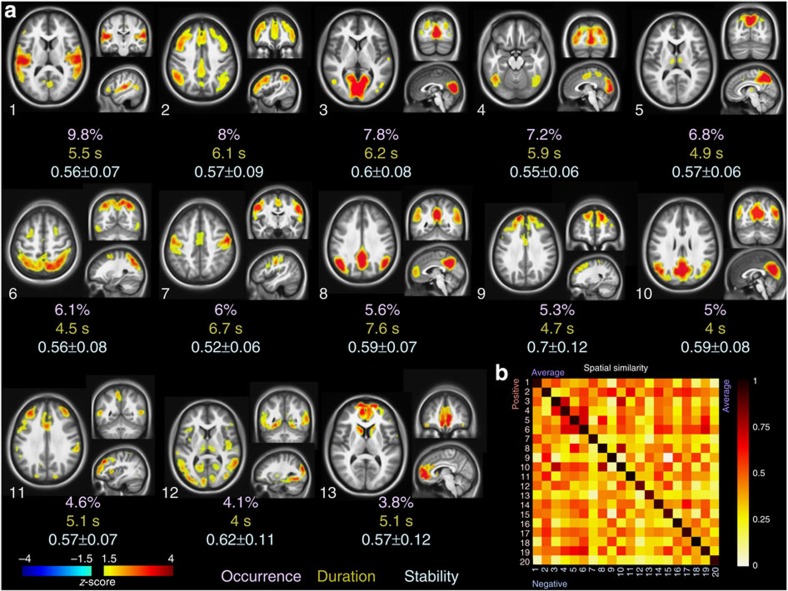
Innovation-driven co-activation patterns (iCAPs). (**a**) We show 13 clusters ordered with respect to their occurrence during resting-state scanning. The stability index is reported as the mean and standard variation of the similarity (cosine distance) between each individual map in the cluster and the average cluster map. Average duration of each cluster is computed by back-projecting each cluster onto the activity-inducing signals. (**b**) Clusters are computed by averaging both positive and negative transients: spatial correlation matrix (bottom right) of positive and negative transient activity maps indicates that the same iCAPs are recovered; that is, high values on the diagonal.

We recovered in total 20 iCAPs that represent consistent maps of transients during rest. Figure 2a shows the top 13 iCAPs ordered with respect to their occurrence rate. The iCAP 1 contains the auditory regions, with high activations in superior and middle temporal gyrus (Heschl gyrus, rolandic operculum) as well as part of the insula, postcentral and precentral gyrus. The iCAP 2 includes regions of the fronto-parietal attention network (ATT) with (superior, middle and inferior) frontal, part of superior(SPL) and inferior parietal lobe (IPL), together with precuneus, anterior cingulate cortex (ACC) and PCC. The iCAP 3 and iCAP 4 encompass primary (calcarine gyrus, cuneus and lingual gyrus) and secondary (middle and inferior occipital, inferior and middle temporal, fusiform, SPL) visual areas, respectively. The iCAP 5 mainly reveals precuneus, PCC and thalamus. The iCAP 6 represents the visuospatial/dorsal attention network comprising IPL, SPL, angular gyrus as well as frontal eye field in middle frontal gyrus^3^,^35^. The iCAP 7 includes the motor network (precentral, postcentral, supplementary motor area (SMA)) and medial frontal gyrus. The iCAP 8 corresponds to the DMN regions with PCC, angular gyrus, parts of precuneus, IPL, middle frontal and medial prefrontal cortex, as well as part of medial temporal lobe. The iCAP 9 covers the anterior executive network with superior frontal gyrus and ACC. The iCAP 10 is the posterior DMN (pDMN), including PCC, IPL, angular, precuneus, as well as visual regions (cuneus). The iCAP 11 shows the anterior salience network with dominant middle and inferior frontal gyrus activations and part of insula^35^. The iCAP 12 is made up of rather diverse regions from limbic and subcortical areas (part of insula, thalamus, hippocampus and parahippocampus), superior and middle temporal gyrus and middle occipital gyrus. The iCAP 13 includes frontal gyrus, ACC and caudate. Spatial maps of all the iCAPs are provided in Supplementary Figs 3 and 4, and details of the brain regions (average *z*-scores and number of voxels in each area) involved in each cluster are provided in Supplementary Table 1.

To confirm the consistency of each iCAP, we computed the stability index as the average similarity distance between the transient activity maps included in each temporal cluster and the cluster's representative iCAP. Temporal clustering of transients does not lead to spatially segregated maps. Spatial similarity between the iCAPs irrespective of the sign of the transients is shown in the upper triangular part of the matrix in Fig. 2b. We also computed the iCAPs separately for positive and negative transients, and show the similarity matrix between these sign-sensitive iCAPs in the lower triangular part (including the diagonal). The high similarity on the diagonal and the strong symmetry of the similarity matrix indicate that the same iCAPs can be recovered from both positive and negative transients, that is, onsets of activations and de-activations carry the same information for clustering.

**Figure 3:**
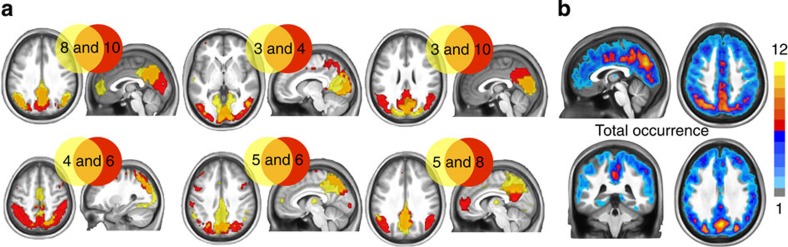
Spatial overlap between iCAPs. (**a**) Six most significant spatially overlapping iCAPs are illustrated. The intersecting areas (in orange) mostly accumulate in the posterior regions; that is, PCC, precuneus, cuneus and superior parietal regions. (**b**) Hub regions are identified by counting the areas that appear consistently in the iCAPs, pointing to precuneus, dorsal PCC, superior parietal lobe, mid cingulate cortex, middle occipital and angular gyrus.

We computed the amount of spatial overlap between iCAPs; the similarity measure is computed by the Jaccard distance, which is the total amount of intersection between two binary patterns (see Methods and Supplementary Fig. 5). We thresholded each iCAP (*z*-score ⩾1.5) to obtain the binary maps. The spatial maps in Fig. 3a illustrate the iCAPs with significant spatial overlap determined by block permutation test (*P*≤0.01 corrected for multiple comparisons, see Supplementary Methods for details). The intersecting areas (in orange) accumulate in the posterior regions; mostly PCC and precuneus (iCAPs 8–10, 5–6, 5–8 and 19–5), cuneus (iCAPs 3–4, 3–10) and superior parietal regions (iCAPs 4–6, 19–6).

To delineate the hub regions of iCAPs, we counted how many times a voxel is present in each thresholded iCAP (*z*-score ⩾1.5). These hub regions, in Fig. 3b, include part of precuneus, dorsal PCC, superior parietal lobe, mid cingulate cortex, middle occipital and angular gyrus.

### Default-mode network de-CAP-sulated

**Figure 4:**
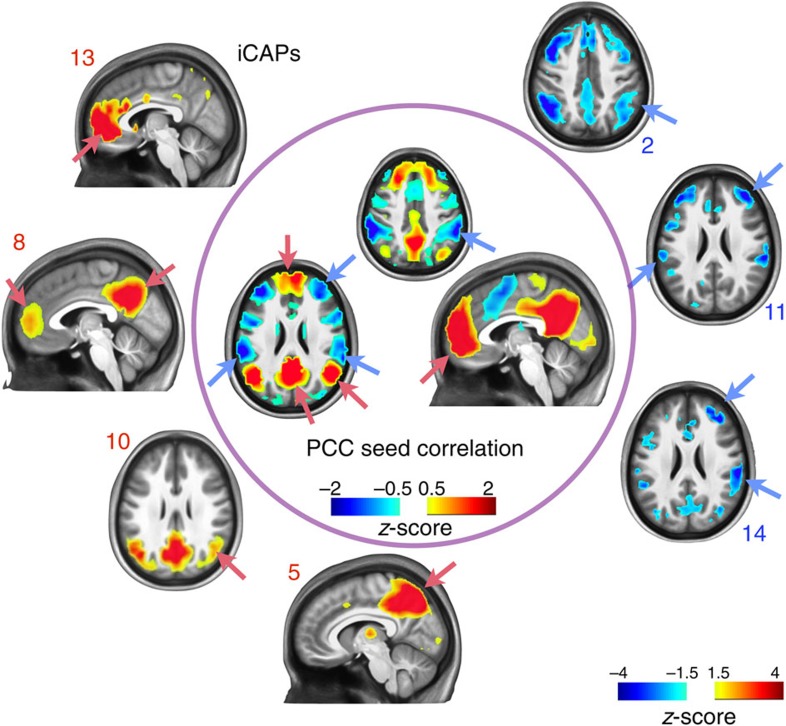
Spatial similarity between the conventional DMN determined by seed–region connectivity analysis with PCC and the iCAPs' spatial maps. Four iCAPs significantly correlate with the positive part of the DMN map, while three iCAPs correlate with the negative part.

We use iCAPs to analyse the conventional DMN determined by seed connectivity analysis. The seed was placed in the PCC, MNI coordinates (0,−53,26), averaged over a 7 × 7 × 11 mm^3^ cubic neighbourhood. The iCAPs that can be associated with the DMN map were established using the cosine distance as a measure of similarity between the DMN map and iCAP maps; contributions to the positive and negative parts of the DMN were computed separately. In particular, in Fig. 4, we show that the iCAPs corresponding to DMN (8), pDMN (10), precuneus (5) and ACC (13) relate to the positive DMN map, whereas attention (2) and anterior salience (11, 14) relate to the negative DMN map, respectively. Spatial correlations of iCAPs with the conventional DMN for the individual subjects are shown in Supplementary Fig. 6.

### Spontaneous activity seen as temporally overlapping iCAPs

We further extracted several dynamical features from the iCAPs representation to characterize their temporal overlap. First, we computed the time course associated with each iCAP by back-projection; that is, iCAP maps are fitted to activity-inducing signals recovered by TA, which is a similar procedure as in ICA. However, the activity-inducing signals are deconvolved from the HRF and de-noised. In addition, positively and negatively constrained weights are obtained by two separate regressions to avoid interactions between iCAPs, and then recombined in a single time course per iCAP as shown in Supplementary Fig. 7. The time courses were normalized by their s.d. and thresholded according to their absolute-valued *z*-score (|z|⩾ 1). We then obtained the duration of all the iCAPs (regardless of their sign) relative to total scanning time. The total on-time of all iCAPs constitute 3.6 times of the total scan duration; that is, for 12 subjects, 350 min of total iCAPs activation out of 96 min of total scanning time. The total on-time was also evenly distributed over the subjects; that is, on average 29±1.8 min of total iCAP activation out of 8 min of resting-state scan per subject. We then computed the average duration of each iCAP individually (Fig. 2a). We found that the DMN (8) has the longest duration (7.6 s), followed by sensory-related iCAPs such as motor (7), visual (3), auditory (1) and attention (2). In terms of occurrence rate of the transients, auditory (1), attention (2) and primary visual (3) are the most common iCAPs.

### iCAPs co-occur in behaviourally relevant combinations

**Figure 5:**
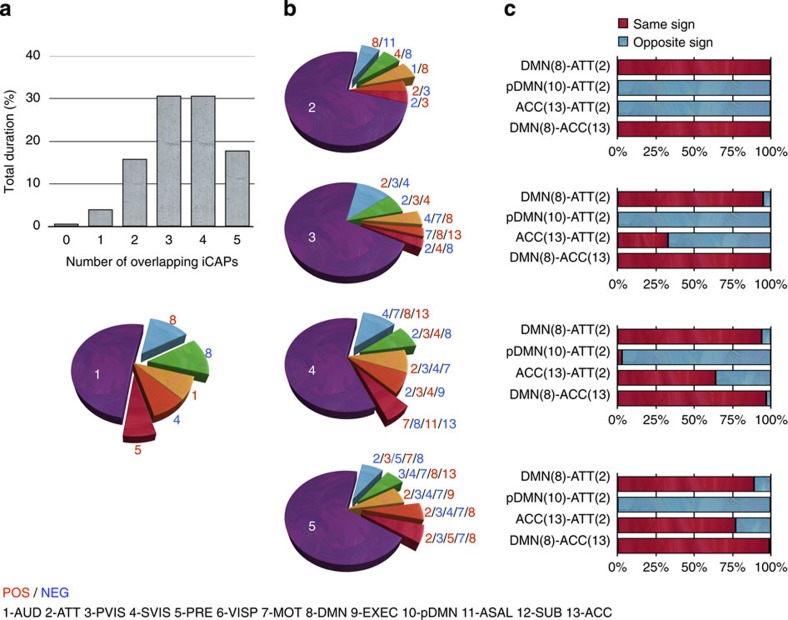
Temporal overlap between the iCAPs. (**a**) The bar plot shows the per cent duration of combinations of overlapping iCAPs. (**b**) For each number of overlapping iCAPs, the pie charts reveal the top five most frequently occurring combinations. DMN-related iCAPs are highlighted. (**c**) The bar plots on the right column depict the percentage of same and opposite signs of combinations that contain both DMN- and attention-related iCAPs for the different sets of overlapping iCAPs. NEG, negative; POS, positive.

To measure the temporal overlap between iCAPs, we counted the different combinations of iCAPs occurring at each time instance. The bar plot in Fig. 5a depicts the percentage of total duration for each number of overlapping iCAPs. On one hand, only 0.7% of the resting-state scan has no active iCAPs, and 4% has only a single iCAP, which is expected given the large total on-time of iCAPs. On the other hand, combinations of iCAPs are most common; that is, two (16%), three (31%), four (31%) and five (18%) iCAPs occurring at the same time account for 95% all together. Despite this significant overlap, not all possible combinations can be observed; that is, while 90% of the iCAPs occur at least once alone, only 58, 29, 15 and 3% of the possible combinations between two, three, four, five iCAPs, respectively, have been registered.

**Figure 6:**
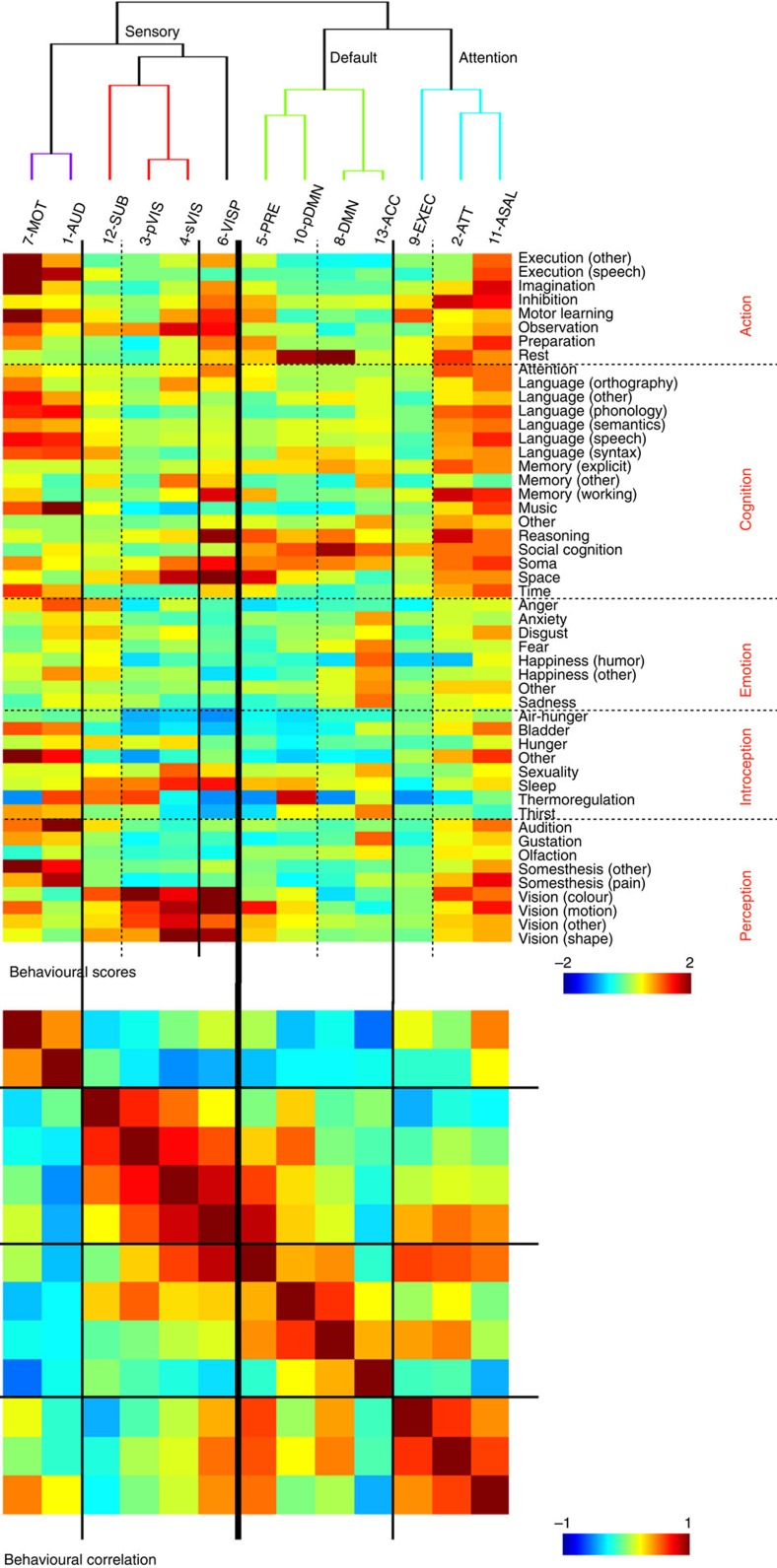
Hierarchical clustering of iCAPs according to their temporal overlap. The dendrogram minimizes the distance at each leaf with respect to the neighbouring leaf, clustering the most similar iCAPs. The behavioural profile of each iCAP is also shown. The highest level of the hierarchy shows groupings into components related to sensory, default mode and attention function. These groups further divide into motor, auditory and visual; posterior default mode and full default mode; executive control and attention networks, respectively. The cross correlation between the iCAPs' behavioural profiles is also consistent with the groupings (bottom).

We then applied hierarchical clustering of iCAPs using the observed combinations as features (in total, 2,098 iCAP combinations were observed out of 55,250 possible iCAPs). To show that this clustering is consistent with putative cognitive processes reflected by iCAPs, we associated each iCAP with its behavioural profile using the Brainmap database^33^. The dendrogram in Fig. 6 reflects the hierarchy of iCAPs together with their behavioural profiles. At the highest level of the hierarchy, iCAPs are grouped as sensory, default-mode and attention networks, respectively. At the same time, the behavioural profiles are also consistent in the same groupings as confirmed by their correlations (Fig. 6); that is, sensory networks show higher scores with their associated role, precuneus (5) and pDMN (10) have both high scores in reasoning and social cognition, DMN (10) and ACC (13) share high scores also in social cognition and explicit memory, whereas ACC (13) alone involves more in emotional processes, finally, attention network involve in both execution and cognition.

### iCAP combinations bring new insight into brain organization

We further analysed the most common combinations of iCAPs by considering the top five for each set of overlapping iCAPs; see Fig. 5b where combinations with DMN-related iCAPs (according to Fig. 4) are disconnected from the pie. The iCAPs that appear most in isolation are DMN (8) in both signs, precuneus (5), auditory (1) and secondary visual (4). The same iCAPs also appear in combination with others. In particular, for two overlapping iCAPs, DMN (8) occurs with anterior salience (11), visual (4), auditory (1) but with opposite signs, and attention (2) overlaps with visual (3) with opposite signs (see Supplementary Fig. 8 for the most frequent 20 iCAP combinations for each set of overlapping iCAPs). With more than two iCAPs, the DMN (8) and ACC (13) show increased overlap when combined with motor, and/or visual iCAPs. Attention (2) further combines with visual (3, 4), and precuneus (5) often occurs in combination with DMN (8) for a large number of overlapping iCAPs. In terms of total on-time, DMN (8) is present ∼38% of the time either alone or in specific combinations with other iCAPs, followed by sensory components such as motor (7; 28%) and auditory (1; 26%). In almost all the combinations, we notice that sensory networks typically occurred with signs opposite to those of attention or DMN-related iCAPs.

We further focused on the specific iCAPs combinations involving both DMN-related (8, 10, 13) and attention iCAPs (2), illustrated by the bar plots in Fig. 5c and ordered with respect to increasing number of overlapping iCAPs from top to bottom. DMN (8) and ATT (2) appear with the same sign more than 95% of the time across all iCAP combinations, whereas pDMN (10) and ATT (2) occur with opposite signs. Another DMN-related iCAP, ACC (13), occurs with ATT (2) with opposite signs when they are combined; however, as they combine with even more iCAPs, there is a reduction in the frequency with which they have opposite signs. Finally, DMN (8) always combines with ACC (13) with the same sign.

## Discussion

In previous work, the TA framework has been validated to recover evoked brain activity without prior knowledge of the stimulation paradigm^32^. Here, we used this framework to reveal transients in spontaneous activity through innovation signals, which encode onsets of activations/de-activations by positive/negative spikes, respectively. The spatial patterns of transients showed clear signatures of known functional networks or subnetworks, even for single transient activity maps without averaging (Fig. 1b,c). Temporal clustering of transient activity maps led to iCAPs that were consistent when selecting only positive, only negative or both types of innovations (Fig. 2b). This indicates that the same spatial patterns can be determined from positive or negative transients; that is, the same regions that jointly activate also de-activate. This suggests that spatial grouping is still a meaningful representation of resting state, even if the sustained activity of different regions is temporally overlapping.

The proposed iCAPs methodology bears similarities with point process analysis with respect to the threshold time course^28^, and with CAPs with respect to the application of temporal clustering^26^,^27^. Direct clustering of (selected) BOLD volumes leads to CAPs that are mixed versions of underlying temporally overlapping activation patterns.

In another recent advance, TFMs^24^ have been applied to recover temporally independent sources. The optimization criterion for temporal independence is applied to BOLD time courses acquired at fast TR, but it is also hindered by temporal overlap. Finally, sliding-window functional connectivity has been used to study non-stationary properties of spatial correlation structure^18^,^19^,^20^,^22^. The main contribution of our work is that TA regularization undoes the effect of hemodynamic blurring and reveals transients of underlying activations. These innovation signals effectively disentangle temporally overlapping activity patterns, and are subsequently clustered in time; that is, transients occurring at different time points can end up in different clusters independent of potential temporal overlap of their underlying sustained activity that was obtained using back-projection of the iCAPs' maps. We provide videos of activity-inducing signals of the TA regularized resting-state data as well as the temporal evolution of iCAPs with their associated behavioural profile of one subject to show how iCAPs dynamically add on top of each other (see Supplementary Videos 1 and 2).

The iCAP maps are spatially well localized and consistent for all iCAP clusters as reflected by their high stability index (Fig. 2). The spatial overlap between the iCAP maps is high (Fig. 3 and Supplementary Fig. 5). Regions that activate consistently in many iCAPs during the resting state are mostly posterior, including dorsal PCC, precuneus, superior parietal lobe, mid cingulate cortex and angular gyrus. Similar findings have previously been reported; that is, the PCC is one of the highly connected hub regions identified by functional connectivity density mapping^36^, and when using it as a seed region of conventional CAP analysis^26^, many different PCC-CAPs are revealed. In general, the spatial overlap between iCAPs points to the fact that many regions co-activate as part of different network configurations at different time points, and thus spatial segregation is not an optimal criterion to study changes in functional networks during resting state (see Supplementary Figs 10 and 11 for a detailed comparison between ICA and iCAP results).

We computed the duration of iCAPs from their sustained-activity signals and counted the time frames during which different combinations of iCAPs are (de-)activated. The total duration of all iCAPs largely exceeds the total scanning time (3.6 times the actual acquisition time), which indicates the high amount of temporal overlap and provides evidence that spontaneous activity is explained by the combinations of three to four iCAPs (Fig. 5a).

Our results provide new insight into the rich dynamic organization of resting state in terms of network components that segregate and integrate over short time intervals. The iCAPs form a compact set of building blocks that can be flexibly combined to describe spontaneous activity fluctuations. The combinations of iCAPs observed are not arbitrary, and we only observed 4% of all possible combinations. Clustering of iCAPs on the basis of these combinations shows a hierarchical organization of large-scale brain networks that is consistent with differences in behaviour profiles of iCAPs; that is, resting-state activity is unravelled into periods when components related to sensory, default-mode and attention, respectively, are dominating (Fig. 6).

The DMN is the hallmark of the brain's resting state and has been referred to as the main hub of the internal mode of cognition, related to higher-level processes such as memory and learning^37^,^38^. Our analysis enables us to study both spatial and temporal interactions of consistent co-activation patterns, including the DMN. The DMN iCAP (8) has the strongest spatial similarity with the conventional DMN as determined by PCC-seed-based connectivity, followed by precuneus (5), pDMN (10) and ACC (13), which represent subnetworks of the DMN; the negative part of the DMN PCC-seed connectivity map correlates with attention (2) and anterior salience (11, 14). Spatial subdivisions can also be obtained for other seed-based connectivity maps, such as primary visual and motor networks (see Supplementary Fig. 12).

The DMN iCAP (8) has the longest average and total duration; that is, it is active for ∼38% of the resting state either alone or in combination with other iCAPs. This suggests that the DMN (8) is the primary building block of resting state and specific iCAPs are consistently co-occurring either with the same or opposite sign (Supplementary Fig. 8). Using seed-based correlation of the PCC, it is commonly reported that activity of DMN is anti-correlated with task-positive networks that include fronto-parietal regions (attention) and ACC (salience)^34^,^39^. In accordance, we found that DMN (8) and salience (11) form the most frequent combination when two iCAPs occur, and consistently with opposite signs. More surprisingly, we also found that DMN (8) and ATT (2) always co-occur with the same signs within any number of iCAP combinations, while pDMN (10) and ATT (2) co-occur with opposite signs (Fig. 5c). These specific iCAP combinations are stable across the subjects (see Supplementary Fig. 13). A handful of recent studies corroborated the positive correlation between DMN and attention networks, for example, during a ‘task-preparation' period^40^ or goal-directed tasks^41^. Moreover, a similar functional association of the attention network and subregions in the cingulate cortex has been suggested in recent literature^25^,^42^,^43^,^44^. In addition, DMN (8) captures more dorsal, and pDMN (10) more ventral, activations in the PCC (Fig. 2), which is in line with subregions of PCC having functionally distinct roles within attention as well as other RSNs^45^. Our work is the first study that clearly demonstrates non-stationary interactions between large-scale brain networks represented by iCAPs; specifically, DMN (8) and pDMN (10) are involved in functionally and dynamically distinct processes.

In sum, spontaneous brain activity measured by fMRI reveals a rich structure of spatially and temporally overlapping functional networks. Extracting transients enables one to become insensitive to temporally overlapping activity and determine consistent spatial patterns at the onset of activation or de-activation. In addition, these patterns can be back-projected to obtain the sustained-activity time course and to quantify temporal features such as occurrence, duration and temporal overlap. The iCAPs view on fMRI resting state reveals how these patterns of brain activity are entangled in space and time, and suggests a more intricate and non-stationary structure than can be revealed using conventional methods. Since iCAPs provide a spatiotemporal zoom onto the DMN, which has been linked to cognitive states^46^,^47^ and neurodegenerative disease^48^,^49^,^50^,^51^, future work should focus on how the organization revealed by iCAPs can advance our understanding of brain function. Another avenue for future research is to identify the electrophysiological correlates of iCAPs. The DMN iCAP played a key role in temporal dynamics, which might relate to previous findings based on spontaneous EEG that DMN has the highest network interaction in the β (14–25 Hz) band^52^. How the different timescales of the fMRI hemodynamic signals (order of seconds) and EEG/MEG electrophysiological signals (order of milliseconds) are bridged remains an open question. One intriguing possibility is that scale-free dynamics of underlying brain activity maintains useful information even after temporal smoothing^53^; notion has been demonstrated for EEG microstates and fMRI RSNs^54^,^55^.

## Methods

### Subjects and procedure

Fourteen healthy volunteers participated in the study (38.4±6 years old). Data acquisitions were obtained with a Siemens 3T Trio TIM scanner, using a 32-channel head coil. The structural images were acquired using a high-resolution three-dimensional T1-weighted Magnetization-Prepared Rapid Gradient-Echo (MPRAGE) sequence (160 slices, TR/TE/FA=2.4 s/2.98 ms/9°, matrix=256 × 240, voxel size=1 × 1 × 1.2 mm^3^). For the resting-state fMRI data, subjects were instructed to lie still and relax in the scanner with their eyes closed. The total acquisition took ∼8 min. The data were acquired using gradient-echo echo-planar imaging (TR/TE/FA=1.1 s/27 ms/90°, matrix=64 × 64, voxel size =3.75 × 3.75 × 5.63 mm^3^, 21 slices, 450 volumes). The first 10 volumes are discarded to assure the magnetization stability.

### FMRI data processing

The fMRI data are preprocessed using custom MATLAB code combined with SPM8 (FIL, UCL, UK) and IBASPM toolboxes^56^. First, fMRI volumes were realigned to the first scan and spatially smoothed with Gaussian filter (full width half maximum=5 mm). We used motion estimation to mark the time points with high frame-wise displacement^57^. Marked frames were not removed as TA relies on the full fMRI time courses to deconvolve, but performed cubic-spline interpolation instead. Two subjects with high motion were excluded from further analysis. The anatomical images are coregistered onto the functional mean image and segmented (NewSegment, SPM8) for the six different MNI templates. The anatomical automatic labelling atlas^58^, composed of 90 regions without the cerebellum, was mapped onto each subject's coregistered anatomical image and further downsampled to match the functional images. The atlasing is only used to guide TA spatial regularization.

### Total activation

The TA framework^32^,^59^ incorporates two main features for fMRI data processing: (1) each voxel's BOLD time course is deconvolved from the temporal blur introduced by the hemodynamic response, leading to the activity-inducing signal that is supposed to show block-type activation patterns (without any prior knowledge on the timing and duration of these blocks); (2) BOLD signals should show a spatial smoothness, which is supposed to be stronger within anatomical atlas regions than across. With that aim, TA solves a convex optimization problem that consists of a least-squares data-fitting term combined with spatial and temporal regularization terms. The temporal regularization uses a differential operator that inverts the hemodynamic-system formulation as in ref. 60, based on the first-order Volterra series approximation of nonlinear Balloon model^61^,^62^. The numerical solution is obtained using generalized forward–backward splitting^63^, which is for the de-noising case also known as parallel Dykstra-like proximal algorithm^64^. TA produces de-noised and well-behaving reconstructions of the activity-related, activity-inducing, and innovation signals.

### Temporal clustering of innovation signals

We thresholded the innovation signals through a two-step procedure; (1) for each subject, we analysed the surrogate data; that is, applied TA on the phase randomized data and performed a subject-wise thresholding (1% confidence interval) and (2) we picked the time points where there are at least 500 ‘active' voxels globally (see Supplementary Figs 1 and 2)^65^. Then, transient activity maps that survive the thresholding are fed into k-means clustering using the cosine distance (see Supplementary Fig. 9).

### iCAPs activation maps and time courses

The iCAPs' spatial maps were computed by combining the clusters of positive and negative (sign-flipped) transients. As the distributions of the maps are not symmetric and one-sided, we subtracted the mode (the maximum value of the histogram) instead of the mean and divided by the s.d. to obtain *z*-scored spatial maps (Fig. 2). The time course of each cluster was computed by back-projecting the iCAPs onto the sustained-activity-inducing signals. The back-projection was computed separately for positive and negative weights to minimize the effect of spatial linear dependency.

### Jaccard distance

We used Jaccard distance to evaluate the amount of spatial overlap between iCAPs activation maps (Supplementary Fig. 5). Jaccard distance is specifically adjusted for binary measurements; that is, X ∈ {0, 1}. We used a threshold |z|>1.5 to obtain binary data for the spatial maps. Jaccard distance (Jd) measures the similarity of two iCAPs by computing the normalized amount of overlap; that is, ratio of intersection to the ratio of union of two iCAPs' spatial maps


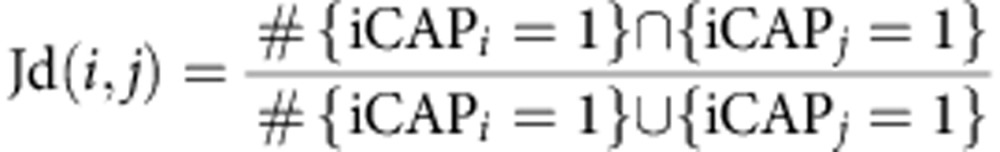


We performed non-parametric testing to detect the significant spatial overlaps between two iCAPs (*P*≤0.01, corrected by maximum statistic). The surrogate data were obtained by spatial block permutation of the binary maps (blocks of size 22 × 37 × 28 mm^3^).

### Spatial correlation with conventional DMN

The conventional DMN was obtained using seed–region correlation; that is, seed was positioned in the PCC: MNI coordinates (0,−53,26), averaged over a 7 × 7 × 11 mm^3^ cubic neighbourhood. The PCC-seed maps were derived for all the subjects and then averaged. We computed the spatial similarity between the iCAPs and group-level conventional DMN using cosine distance as the similarity measure (Fig. 4). The subject-level similarities are shown in Supplementary Fig. 6.

### Temporal overlap

The total and average durations of iCAPs were computed from the normalized iCAPs' time courses. Specifically, at each time point, we counted the number of ‘active' iCAPs regardless of the sign of the weight, and observed the distribution over number of iCAPs versus total time. We then specified which iCAP combinations occur mostly for each number of overlap (see Fig. 5b and Supplementary Fig. 8). We have further investigated iCAP combinations that relate to default-mode and attention. For each number of iCAPs combinations, from two to five iCAP combinations, we explored their functional interactions by comparing their activation signs. (see Fig. 5c). We investigated whether these combinations are consistent across subjects by measuring the mean total duration and standard error of these specific iCAP combinations (see Supplementary Fig. 13).

## Additional information

**How to cite this article:** Karahanoğlu, F. I. & Van De Ville, D. Transient brain activity disentangles fMRI resting-state dynamics in terms of spatially and temporally overlapping networks. *Nat. Commun.* 6:7751 doi: 10.1038/ncomms8751 (2015).

## Supplementary Material

Supplementary Information Supplementary Figures 1-13, Supplementary Table 1, Supplementary Methods and Supplementary References

Supplementary Movie 1 Activity-inducing signals followed by innovation signals of the TA regularized resting-state data for one subject

Supplementary Movie 2 Temporal evolution of innovation driven co-activation patterns (iCAPs) with their associated behavioral profile of one subject
