# Supplementary material for: Transient brain activity disentangles fMRI resting-state dynamics in terms of spatially and temporally overlapping networks
Source: Nat Commun. 2015 Jul 16;6:7751. doi: 10.1038/ncomms8751 (PMC4518303; doi:10.1038/ncomms8751)
Supplement: Supplementary Information — Supplementary Figures 1-13, Supplementary Table 1, Supplementary Methods and Supplementary References [file ncomms8751-s1.pdf]

Supplementary Figure 1. Histograms of original and phase-randomised data

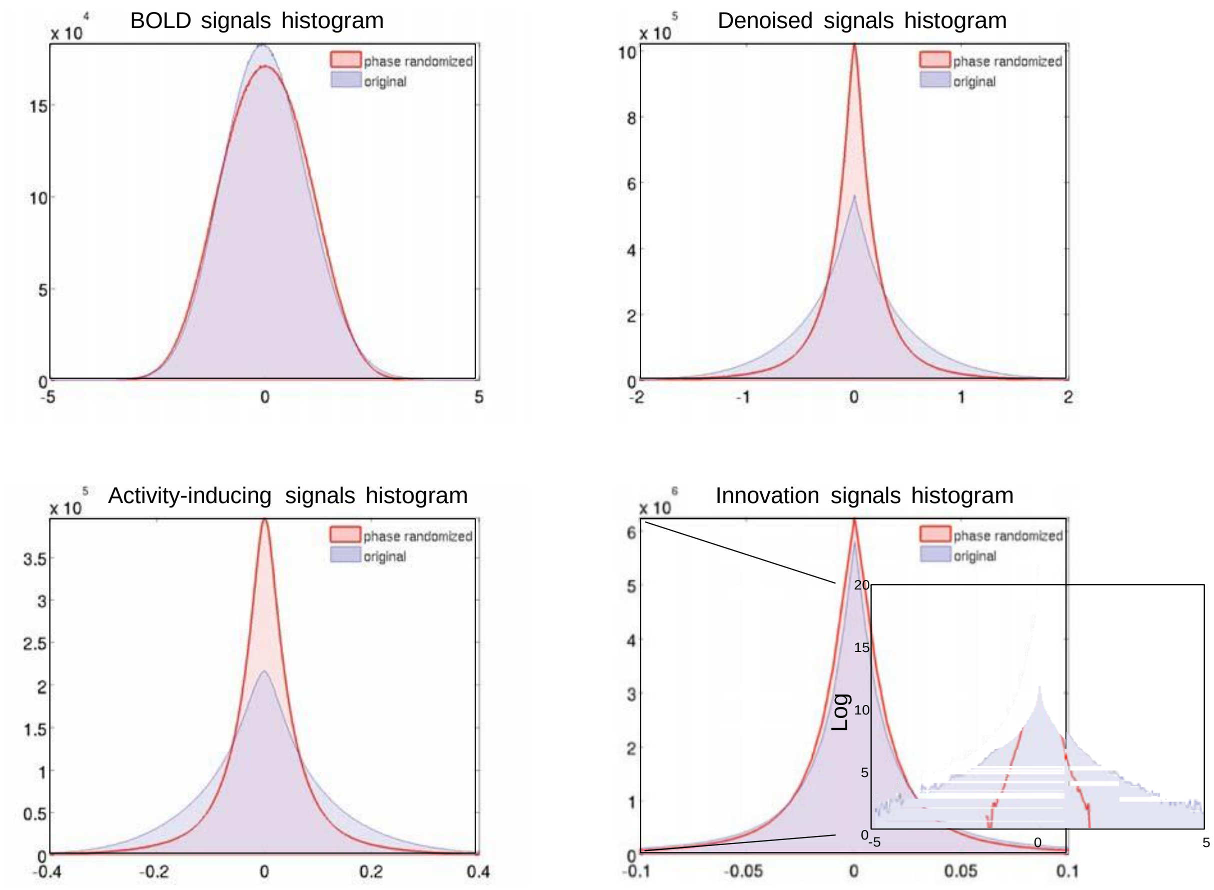

Figure S1: Comparison of histograms before and after applying TA for real and phase-randomized data.

Supplementary Figure 2. Subject and group-level thresholding for detecting the transients

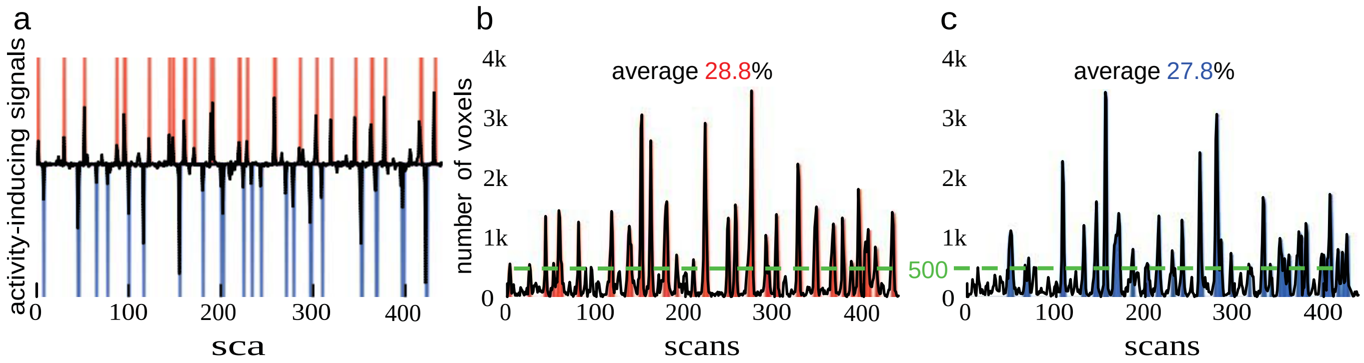

Figure S2: (a) Subject-wise thresholding of innovations for a voxel time course, the threshold is determined by the surrogate data (1% confidence interval). (b) the group-wise thresholding (500 voxels) of positive and (c) negative innovations, which constitute 28% and 27% of all time points (5280), respectively.

Supplementary Figure 3. Spatial iCAP maps

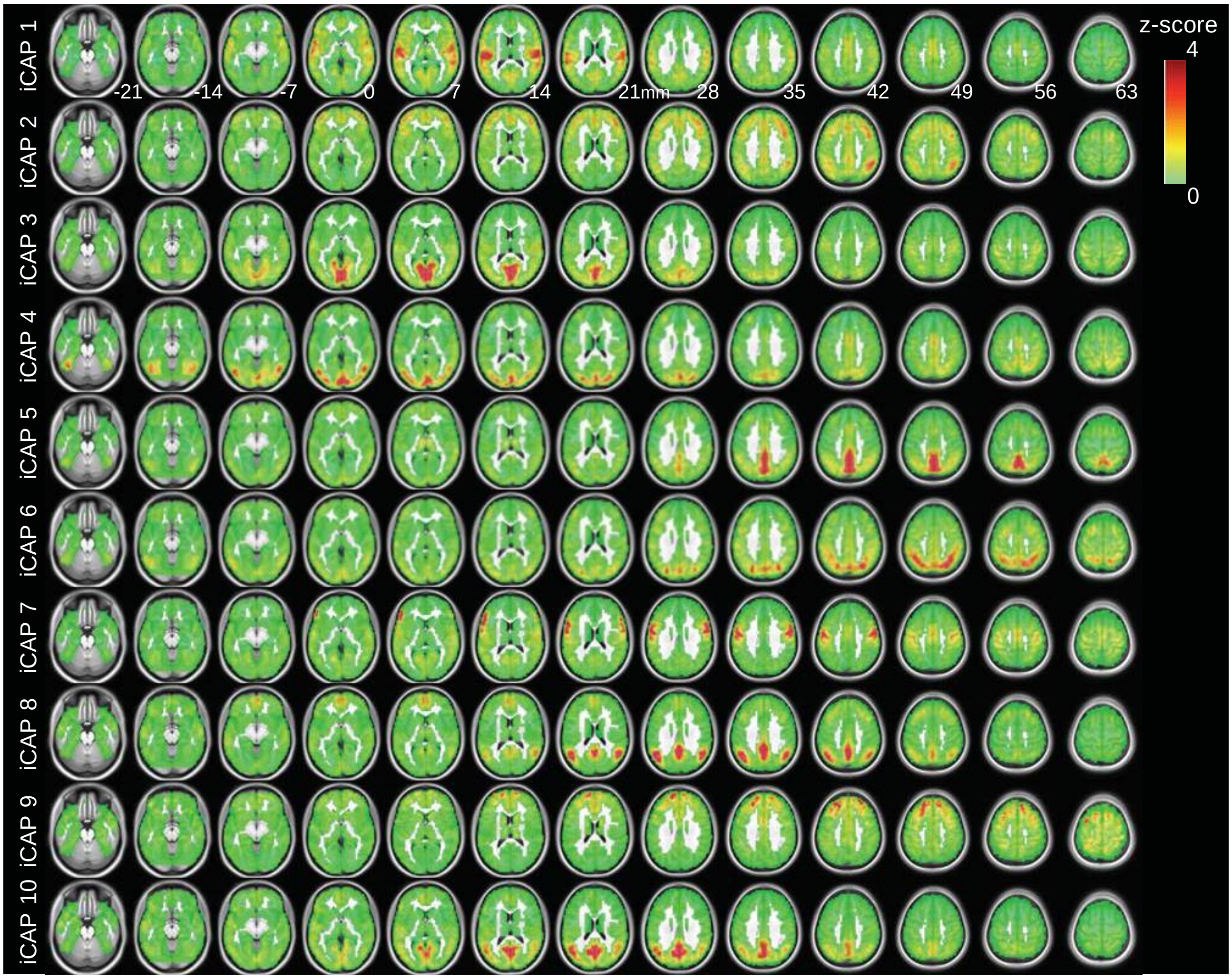

Figure S3: iCAPs 1–10

Supplementary Figure 4. Spatial iCAP maps

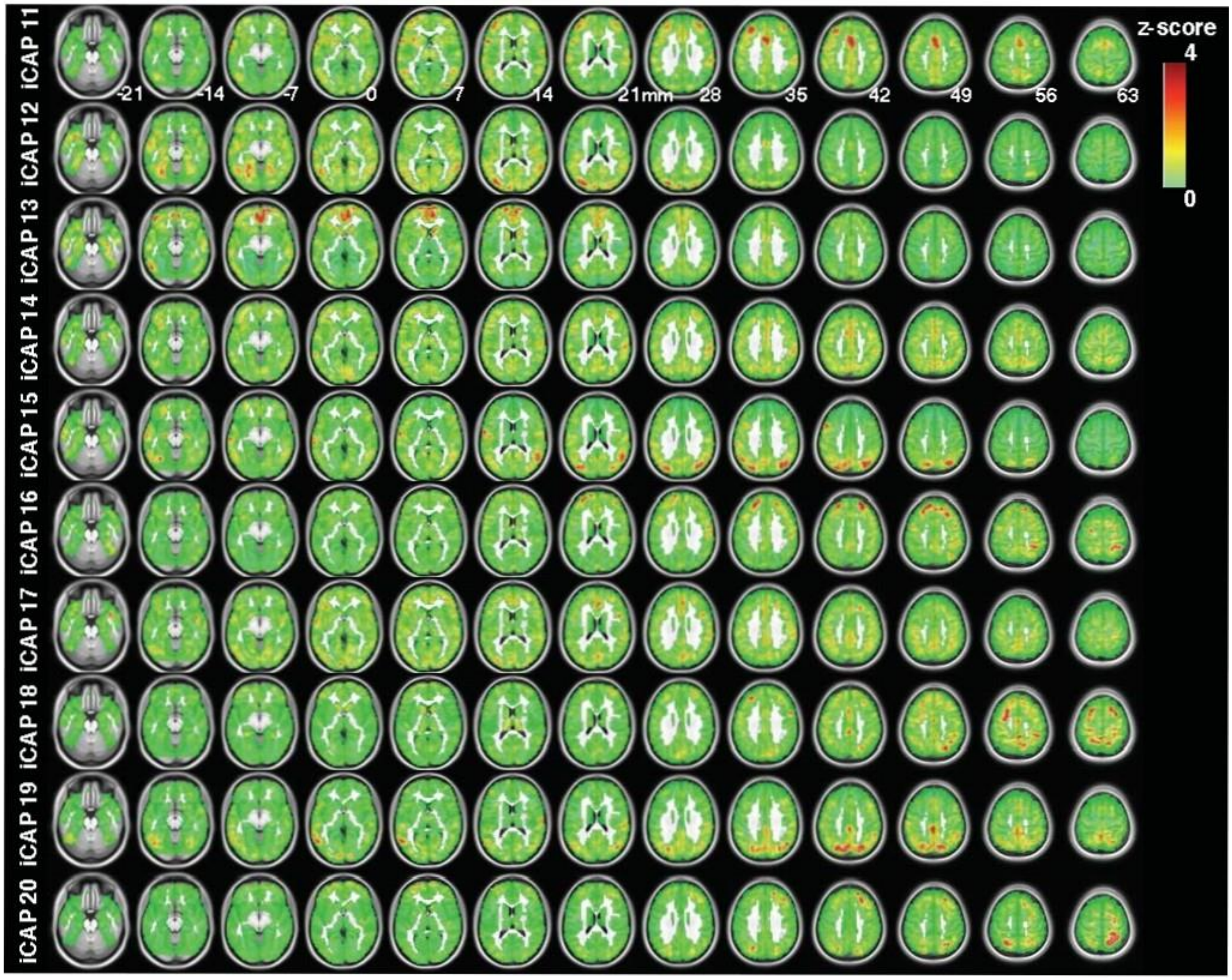

Figure S4: iCAPS 11–20

Supplementary Figure 5. Amount of spatial and temporal overlap between iCAPs

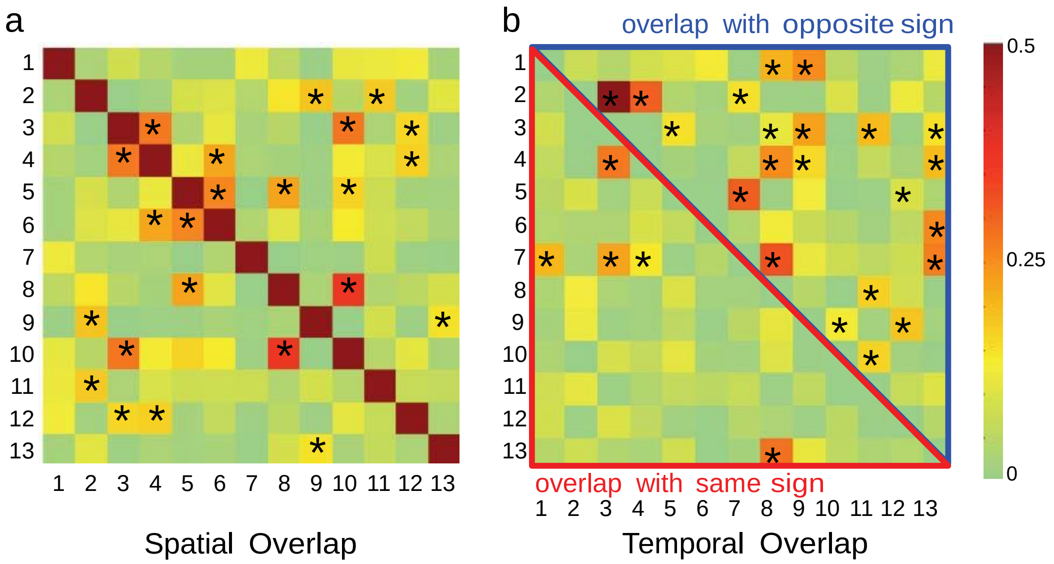

Figure S5: Spatial and temporal overlap of iCAPs. We used Jaccard's distance to evaluate the amount of spatial and temporal overlap of each iCAP. The temporal overlap is computed for same signed and opposite signed activations. The stars indicate significant interactions through non-parametric test ($p < 0.05$ corrected for multiple comparisons).

Supplementary Figure 6. Spatial resemblance of subject-specific DMN with iCAPs

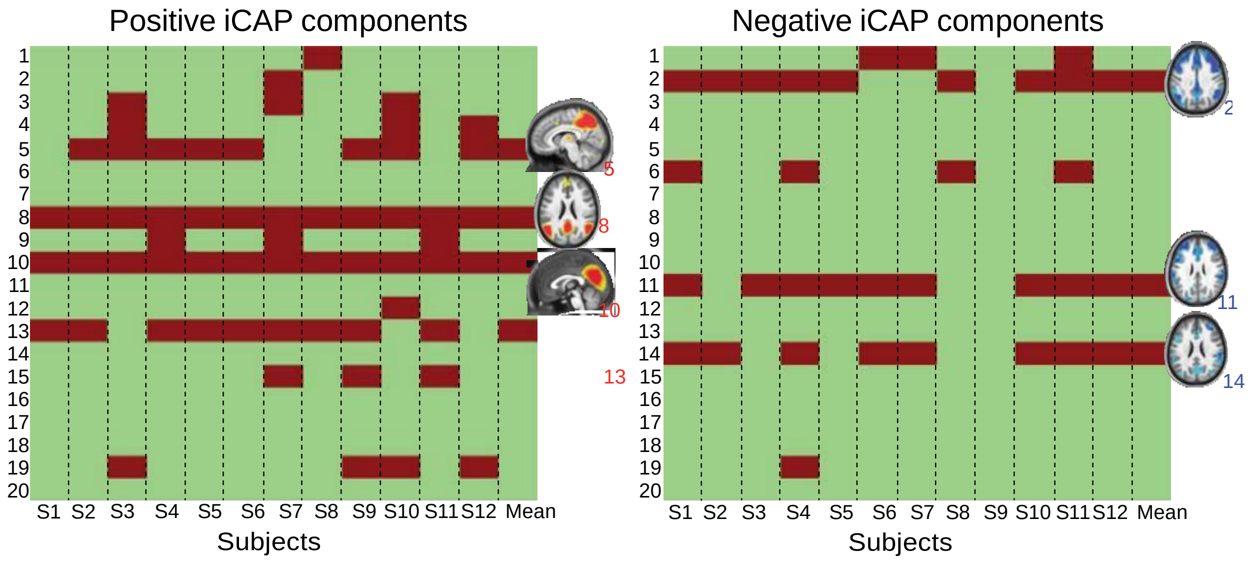

Figure S6: Spatial correlation of subject-wise PCC seed networks with iCAPs.

Supplementary Figure 7. iCAPs' sustained-activity time courses

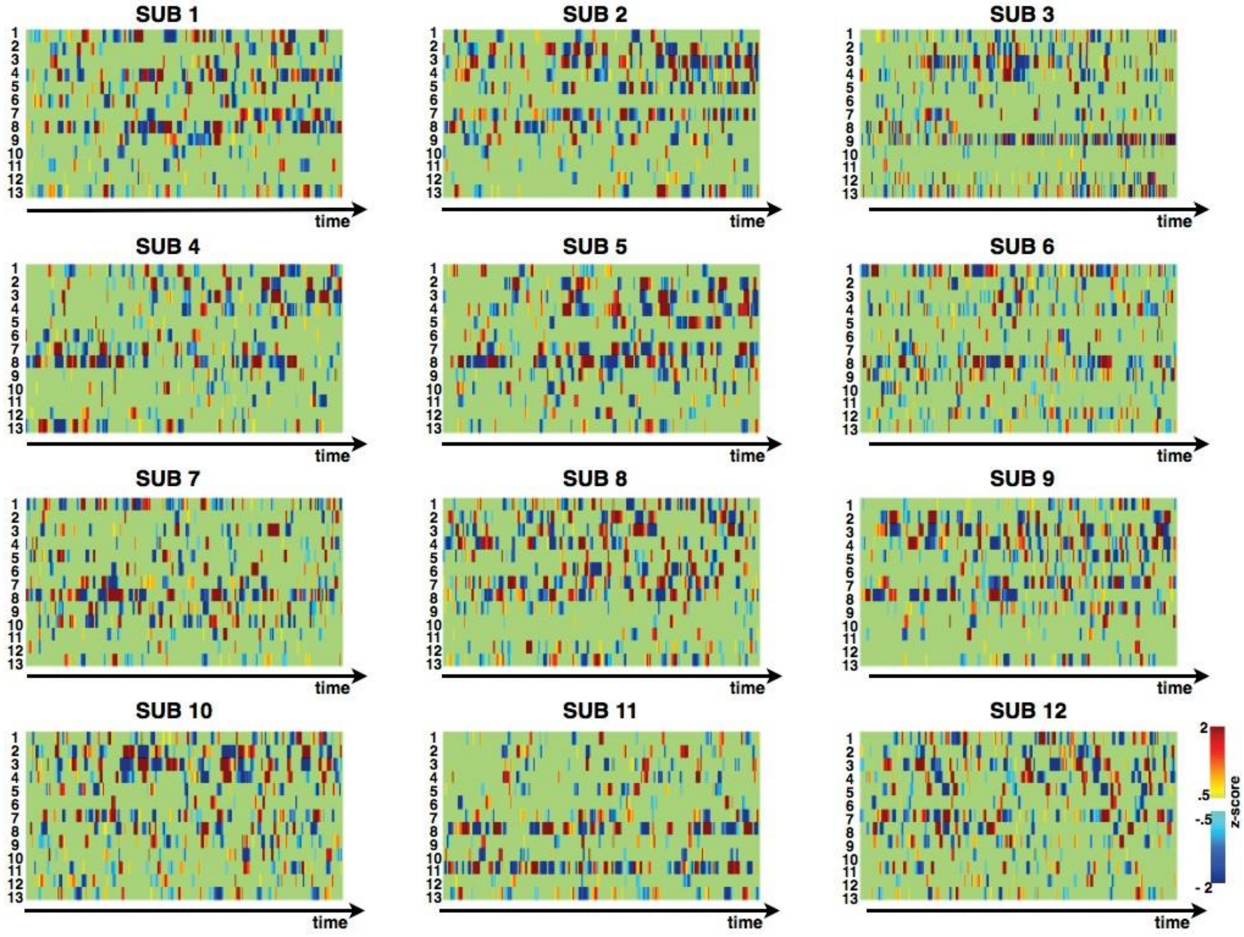

Figure S7: iCAPs' sustained-activity time courses for each subject.

Supplementary Figure 8. Most-frequent iCAP combinations for different hierarchy levels

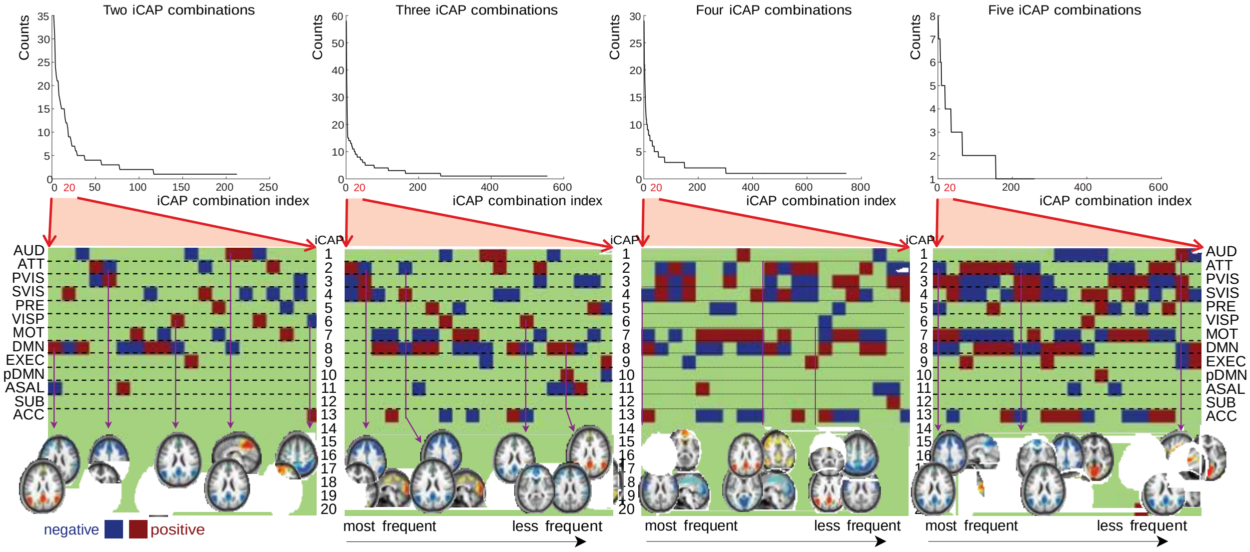

Figure S8: Combinations of 20 most-frequent iCAPs occurring in different number of temporal overlap. Red and blue corresponds to positive and negative state, respectively.

Supplementary Figure 9. Selecting the number of clusters

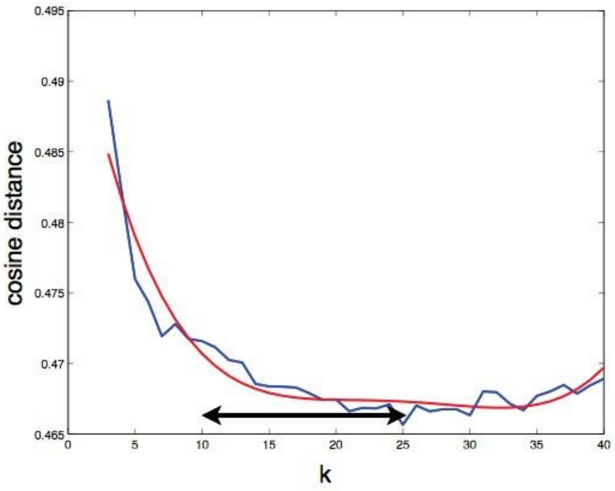

Figure S9: Average out-of-fold cost for the k-mean clustering (blue) with its polynomial fit (red). There is a range of reasonable values for the number of clusters (indicated by the arrow). We opted for 20 clusters, but limited the detailed analysis to 13.

Supplementary Figure 10. Comparison of ICA and iCAPs in the spatial domain

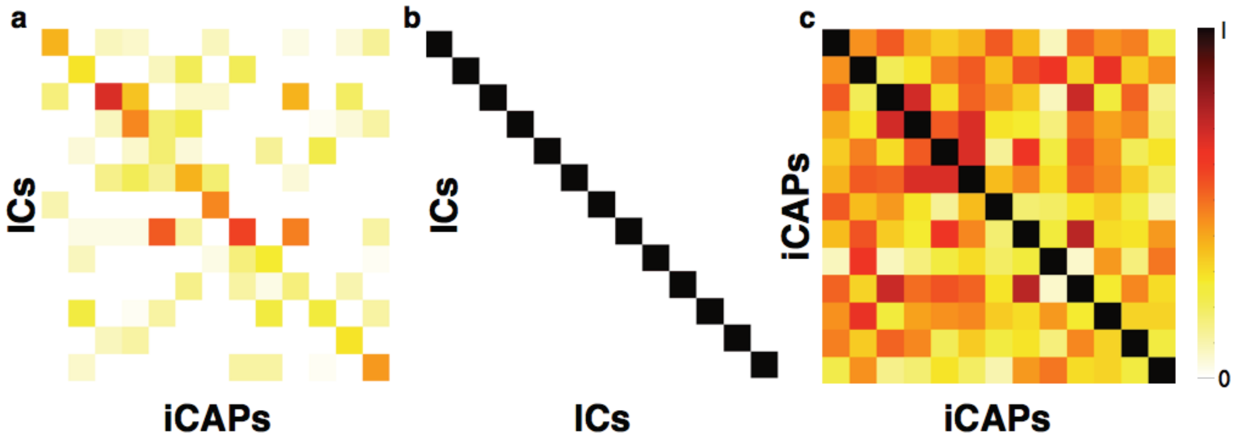

Figure S10: Comparison of ICA and iCAPs in the spatial domain. (a) Spatial similarity between iCAPs and matched ICs. (b) Spatial similarity between ICs. (c) Spatial similarity between iCAPs.

Supplementary Figure 11. Comparison of ICA and iCAPs in the temporal domain

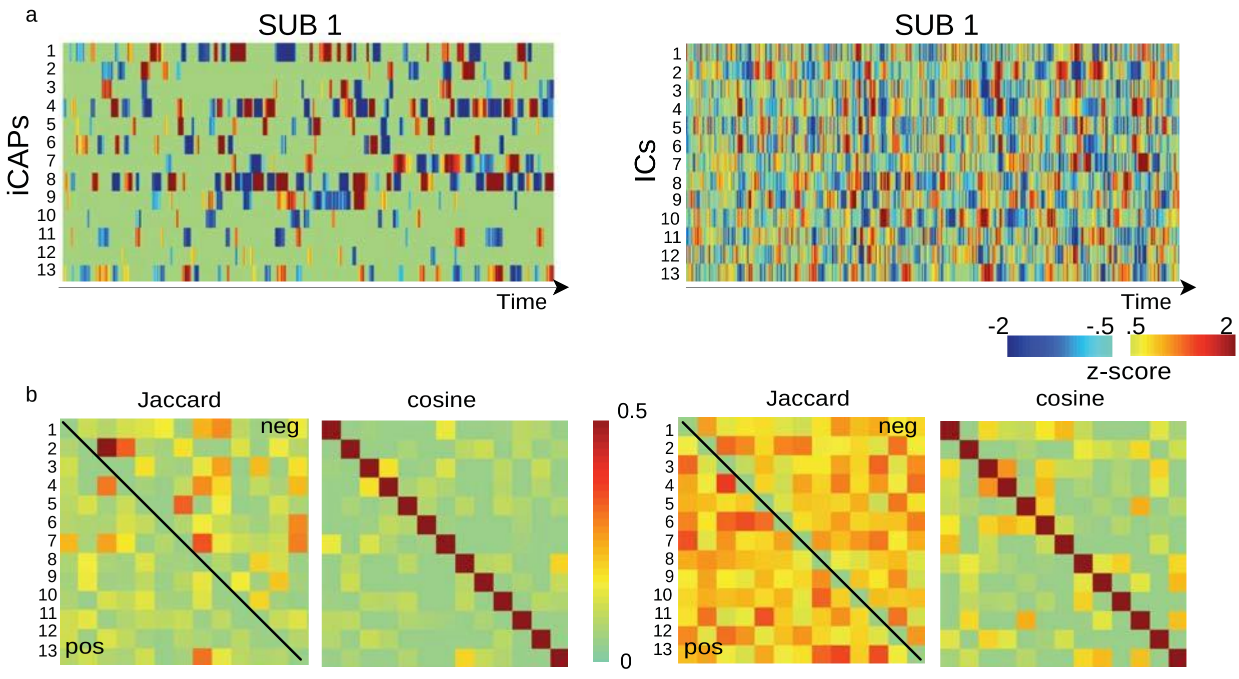

Figure S11: Comparison of ICA and iCAPs in the temporal domain. Results are shown for a typical subject. Left are the ICAPs results; right the ICA ones. (a) Timecourses. (b) Temporal similarity between iCAPs and IC timecourses according to Jaccard distance and cosine distance.

Supplementary Figure 12. Subdivision of seed based connectivity maps of primary visual and motor by iCAPs

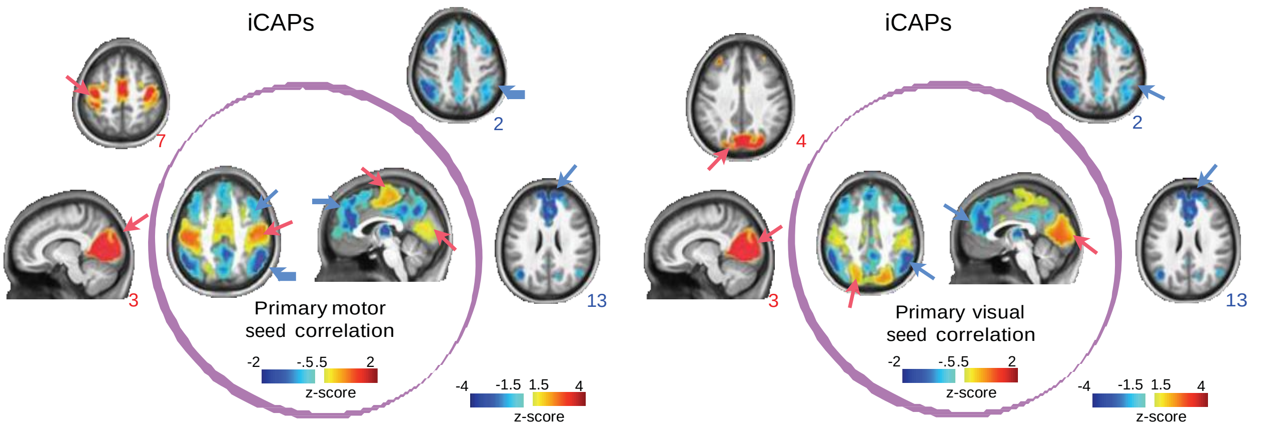

Figure S12: Average out-of-fold cost for the k-mean clustering (blue) with its polynomial fit (red). There is a range of reasonable values for the number of clusters (indicated by the arrow). We opted for 20 clusters, but limited the detailed analysis to 13.

Supplementary Figure 13. The mean duration and standard error of iCAPs combinations including both pDMN (10) and ATT(2), and DMN (8) and ATT (2) over the subjects

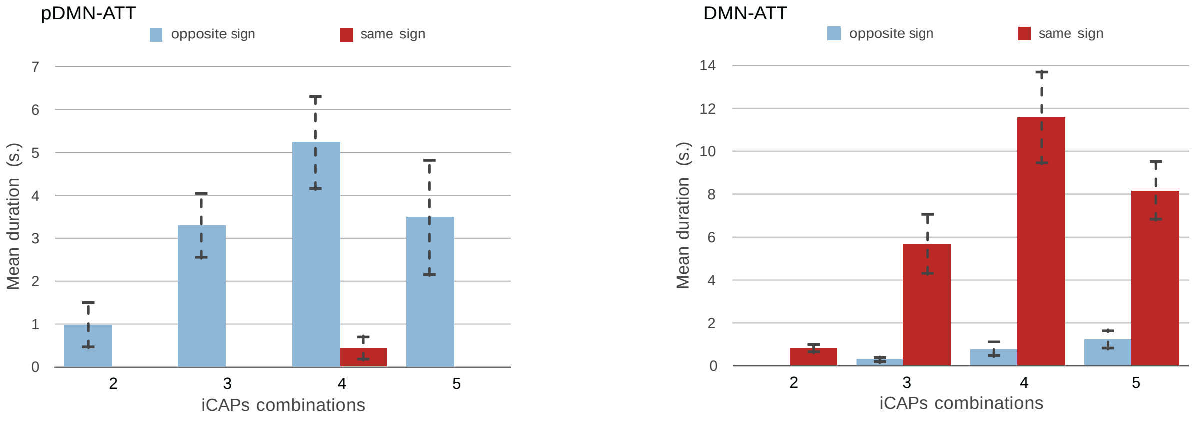

Figure S13: The mean total duration of iCAP combinations (with standard error across subjects) including both pDMN (10) and ATT (2) on the left, and DMN (10) and ATT (2) on the right. pDMN and ATT activates with opposite signs, whereas DMN and ATT activates with the same sign in almost all numbers of iCAP combinations.

Supplementary Table 1. List of regions in each iCAP

iCAP	Region	Lobe	Z-value	voxels	iCAP	Region	Lobe	Z-value	voxels
1	Transverse Temporal Gyrus	Temporal	3.68	40	12	Hippocampus	Temporal	2.17	15
	Sup Temporal Gyrus	Temporal	2.66	393		Lingual Gyrus	Occipital	2.16	69
	Postcentral Gyrus	Frontal	2.63	102		Cingulate Gyrus	Limbic	2.11	25
	Insula	Sub-lobar	2.24	177		Parahippocampal Gyrus	Limbic	2.1	77
	Inf Par Lobule	Parietal	2.19	85		Insula	Sub-lobar	1.99	89
	Mid Temporal Gyrus	Temporal	2.06	184		Sup Temporal Gyrus	Temporal	1.97	204
	Precentral Gyrus	Frontal	2.05	99		Sup Par Lobule	Parietal	1.97	27
	Supramarginal Gyrus	Parietal	1.92	38		Inf Occipital Gyrus	Occipital	1.94	14
	Paracentral Lobule	Frontal	1.79	18		Precuneus	Parietal	1.93	42
	Med Frontal Gyrus	Frontal	1.77	31		Putamen	Sub-lobar	1.89	27

Table 1 – continued from previous page

iCAP	Region	Lobe	Z-value	voxels	iCAP	Region	Lobe	Z-value	voxels
	Mid Frontal Gyrus 6	Frontal	1.77	10		Inf Frontal Gyrus	Frontal	1.85	106
	Precuneus	Parietal	1.75	49		Precentral Gyrus 4	Frontal	1.85	11
	Cingulate Gyrus	Limbic	1.74	42		Thalamus	Sub-lobar	1.81	31
	Posterior Cingulate	Limbic	1.74	36		Posterior Cingulate	Limbic	1.81	38
	Inf Frontal Gyrus	Frontal	1.72	109		Ant Cingulate	Limbic	1.79	11
	Cuneus	Occipital	1.69	67		Transverse Temporal Gyrus	Temporal	1.74	14
	Inf Par Lobule	Parietal	2.23	223		Supramarginal Gyrus	Parietal	1.73	23
	Mid Frontal Gyrus	Frontal	2.13	608		Med Frontal Gyrus	Frontal	2.97	234
	Precentral Gyrus	Frontal	2.02	47		Caudate	Sub-lobar	2.79	85
**2**	Med Frontal Gyrus	Frontal	1.97	183		Ant Cingulate	Limbic	2.76	140
	Inf Frontal Gyrus	Frontal	1.94	206		Sup Frontal Gyrus	Frontal	2.75	186

13

Table 1 – continued from previous page

iCAP	Region	Lobe	Z-value	voxels	iCAP	Region	Lobe	Z-value	voxels
	Sup Frontal Gyrus	Frontal	1.93	321		Fusiform Gyrus	Temporal	2.45	38
	Supramarginal Gyrus	Parietal	1.92	25		Inf Temporal Gyrus	Temporal	2.32	46
	Cingulate Gyrus	Limbic	1.91	102		Amygdala	Limbic	2.19	27
	Ant Cingulate	Limbic	1.85	68		Mid Frontal Gyrus	Frontal	2.17	95
	Angular Gyrus	Parietal	1.85	21		Thalamus	Sub-lobar	2.15	4
	Sup Par Lobule	Parietal	1.85	46		Sub-Gyral 7	Frontal	2.14	6
	Precuneus	Parietal	1.65	71		Inf Frontal Gyrus 5	Frontal	2.12	70
**3**	Lingual Gyrus	Occipital	3.25	223		Mid Occipital Gyrus	Occipital	2.07	24
	Posterior Cingulate	Limbic	2.96	98		Mid Temporal Gyrus	Temporal	2.03	118
	Cuneus	Occipital	2.91	337		Cingulate Gyrus	Limbic	2	44
	Precuneus	Parietal	2.34	101		Inf Occipital Gyrus	Occipital	1.96	12
	Parahippocampal Gyrus	Limbic	2.09	19		Sup Temporal Gyrus	Temporal	1.95	24

Table 1 – continued from previous page

iCAP	Region	Lobe	Z-value	voxels	iCAP	Region	Lobe	Z-value	voxels
	Fusiform Gyrus	Occipital	1.74	23		Precentral Gyrus	Frontal	1.66	13
	Mid Occipital Gyrus	Occipital	1.73	65		Precuneus	Parietal	1.64	18
	Postcentral Gyrus	Parietal	1.7	29		Amygdala	Limbic	2.36	8
	Mid Temporal Gyrus	Temporal	1.65	59		Parahippocampal Gyrus	Limbic	2.13	19
	Sup Temporal Gyrus	Temporal	1.6	22		Inf Temporal Gyrus	Temporal	2.05	15
	Lingual Gyrus	Occipital	2.87	135		Sup Par Lobule	Parietal	2.04	77
	Cuneus	Occipital	2.69	314		Inf Par Lobule	Parietal	2.04	128
	Mid Occipital Gyrus	Occipital	2.59	243		Cingulate Gyrus	Limbic	2.02	138
	Fusiform Gyrus	Temporal	2.48	87		Supramarginal Gyrus	Parietal	2	31
**4**	Inf Occipital Gyrus	Occipital	2.41	46	**14**	Sup Frontal Gyrus	Frontal	1.98	152
	Mid Temporal Gyrus	Temporal	2.12	74		Sub-Gyral	Frontal	1.97	11
	Sup Occipital Gyrus	Occipital	2.11	11		Mid Frontal Gyrus	Frontal	1.97	216

Table 1 – continued from previous page

iCAP	Region	Lobe	Z-value	voxels	iCAP	Region	Lobe	Z-value	voxels
	Med Frontal Gyrus	Frontal	1.92	40		Mid Temporal Gyrus	Temporal	1.96	89
	Cingulate Gyrus	Limbic	1.91	37		Precuneus	Parietal	1.95	247
	Sup Par Lobule	Parietal	1.88	66		Med Frontal Gyrus	Frontal	1.92	120
	Precuneus	Parietal	1.85	170		Postcentral Gyrus	Parietal	1.92	90
	Paracentral Lobule	Frontal	1.75	50		Lingual Gyrus	Occipital	1.9	80
	Postcentral Gyrus	Parietal	1.73	48		Inf Frontal Gyrus	Frontal	1.88	132
	Precuneus	Parietal	3.51	412		Cuneus	Occipital	1.79	89
	Cingulate Gyrus	Limbic	2.87	108		Mid Occipital Gyrus	Occipital	1.76	25
	Cuneus	Occipital	2.53	49		Sup Temporal Gyrus	Temporal	1.76	85
**5**	Paracentral Lobule	Frontal	2.38	55		Precentral Gyrus	Frontal	1.76	106
	Postcentral Gyrus	Parietal	2.17	32		Ant Cingulate	Limbic	1.75	36
	Sup Par Lobule	Parietal	2.11	115		Insula	Sub-lobar	1.73	17

Table 1 – continued from previous page

iCAP	Region	Lobe	Z-value	voxels
	Posterior Cingulate	Limbic	1.97	12
	Thalamus	Sub-lobar	1.97	30
	Inf Par Lobule	Parietal	1.79	99
	Supramarginal Gyrus	Parietal	1.75	10
	Fusiform Gyrus	Occipital	1.74	16
	Mid Temporal Gyrus	Temporal	1.63	13
	Inf Temporal Gyrus	Occipital	1.53	10
	Sup Par Lobule	Parietal	2.96	221
	Precuneus	Parietal	2.48	393
	Inf Par Lobule	Parietal	2.36	254
	Sup Occipital Gyrus	Occipital	2.33	19
6	Cuneus	Occipital	2.26	129

iCAP	Region	Lobe	Z-value	voxels
	Thalamus	Sub-lobar	1.69	9
	Paracentral Lobule	Frontal	1.69	8
	Posterior Cingulate	Limbic	1.65	29
	Fusiform Gyrus	Occipital	1.63	10
	Sup Occipital Gyrus	Occipital	3.21	34
	Precuneus	Parietal	2.76	242
	Angular Gyrus	Parietal	2.73	36
	Parahippocampal Gyrus	Limbic	2.71	15
	Sup Par Lobule	Parietal	2.64	69
15	Sup Temporal Gyrus	Temporal	2.49	102
	Supramarginal Gyrus	Parietal	2.42	37
	Mid Temporal Gyrus	Temporal	2.39	224

Table 1 – continued from previous page

iCAP	Region	Lobe	Z-value	voxels	iCAP	Region	Lobe	Z-value	voxels
	Postcentral Gyrus	Parietal	1.91	90		Cuneus	Occipital	2.32	60
	Mid Frontal Gyrus	Frontal	1.85	76		Inf Temporal Gyrus	Temporal	2.31	47
	Mid Occipital Gyrus	Occipital	1.84	80		Precentral Gyrus	Frontal	2.29	36
	Paracentral Lobule	Frontal	1.82	23		Mid Occipital Gyrus	Occipital	2.22	66
	Inf Frontal Gyrus	Frontal	1.81	15		Mid Frontal Gyrus	Frontal	2.19	76
	Inf Temporal Gyrus	Occipital	1.8	30		Posterior Cingulate	Limbic	2.08	29
	Mid Temporal Gyrus	Temporal	1.75	54		Inf Par Lobule	Parietal	2.07	115
	Lingual Gyrus	Occipital	1.74	34		Fusiform Gyrus	Temporal	2.04	20
	Precentral Gyrus	Frontal	1.73	19		Postcentral Gyrus	Parietal	1.99	40
	Fusiform Gyrus	Temporal	1.72	28		Inf Frontal Gyrus 4	Frontal	1.97	30
	Sup Frontal Gyrus	Frontal	1.71	26		Insula	Sub-lobar	1.64	10
	Cingulate Gyrus	Limbic	1.67	5		Lingual Gyrus	Occipital	1.63	17

Table 1 – continued from previous page

iCAP	Region	Lobe	Z-value	voxels	iCAP	Region	Lobe	Z-value	voxels
	Inf Frontal Gyrus	Frontal	4.11	128		Postcentral Gyrus	Parietal	3.22	90
	Precentral Gyrus	Frontal	3.24	321		Sup Frontal Gyrus	Frontal	3.13	261
	Postcentral Gyrus	Parietal	2.15	176		Sup Par Lobule	Parietal	2.87	48
**7**	Mid Frontal Gyrus	Frontal	2.02	46		Precentral Gyrus	Frontal	2.66	111
	Med Frontal Gyrus	Frontal	1.96	79	**16**	Mid Temporal Gyrus	Temporal	2.55	12
	Inf Par Lobule	Parietal	1.87	30		Inf Par Lobule	Parietal	2.53	52
	Paracentral Lobule	Frontal	1.78	15		Mid Frontal Gyrus	Frontal	2.37	225
	Sup Temporal Gyrus	Temporal	1.77	24		Cuneus	Occipital	2.28	15
	Transverse Temporal Gyrus	Temporal	1.75	17		Inf Frontal Gyrus	Frontal	2.14	42
	Insula	Sub-lobar	1.74	10		Caudate	Sub-lobar	2.12	16
	Cingulate Gyrus	Limbic	1.67	20		Med Frontal Gyrus	Frontal	2.06	22

Table 1 – continued from previous page

iCAP	Region	Lobe	Z-value	voxels	iCAP	Region	Lobe	Z-value	voxels
	Posterior Cingulate	Limbic	1.65	13		Sub-Gyral	Frontal	2.05	10
	Angular Gyrus	Parietal	4.04	41		Sup Temporal Gyrus	Temporal	1.96	10
	Sup Temporal Gyrus	Temporal	3.22	82		Mid Occipital Gyrus	Occipital	1.96	6
	Precuneus	Parietal	3.15	273		Precuneus	Parietal	1.91	46
	Mid Temporal Gyrus	Temporal	2.98	118		Ant Cingulate	Limbic	1.86	13
**8**	Cingulate Gyrus	Limbic	2.92	77		Cingulate Gyrus	Limbic	1.8	17
	Posterior Cingulate	Limbic	2.86	74		Mid Occipital Gyrus	Occipital	2.23	122
	Inf Par Lobule	Parietal	2.81	109		Sup Temporal Gyrus	Temporal	2.21	117
	Cuneus	Occipital	2.6	16		Ant Cingulate	Limbic	2.19	74
	Supramarginal Gyrus	Parietal	2.58	45		Inf Frontal Gyrus	Frontal	2.14	133
	Sup Occipital Gyrus	Occipital	2.43	11		Cuneus	Occipital	2.13	118
	Sup Par Lobule	Parietal	2.13	33		Cingulate Gyrus	Limbic	2.07	118
					**17**				

Table 1 – continued from previous page

iCAP	Region	Lobe	Z-value	voxels	iCAP	Region	Lobe	Z-value	voxels
	Med Frontal Gyrus	Frontal	2.11	133		Mid Frontal Gyrus	Frontal	2.05	117
	Sup Frontal Gyrus	Frontal	1.78	43		Inf Par Lobule	Parietal	2.04	153
	Ant Cingulate	Limbic	1.76	28		Fusiform Gyrus	Temporal	2.04	24
	Mid Frontal Gyrus	Frontal	1.67	15		Mid Temporal Gyrus	Temporal	2.03	57
	Sup Frontal Gyrus	Frontal	2.63	565		Med Frontal Gyrus	Frontal	2	112
	Mid Frontal Gyrus	Frontal	2.29	337		Precentral Gyrus	Frontal	1.98	105
**9**	Thalamus	Sub-lobar	2.13	5		Inf Occipital Gyrus	Occipital	1.98	21
	Postcentral Gyrus	Parietal	2.12	110		Precuneus	Parietal	1.95	145
	Caudate	Sub-lobar	2.11	19		Inf Temporal Gyrus	Occipital	1.92	23
	Sup Par Lobule	Parietal	2	16		Lingual Gyrus	Occipital	1.91	70
	Precentral Gyrus	Frontal	1.91	65		Insula	Sub-lobar	1.9	19
	Med Frontal Gyrus	Frontal	1.89	166		Postcentral Gyrus	Parietal	1.88	75

Table 1 – continued from previous page

iCAP	Region	Lobe	Z-value	voxels
	Ant Cingulate	Limbic	1.86	46
	Cingulate Gyrus	Limbic	1.77	73
	Inf Frontal Gyrus	Frontal	1.75	19
**10**	Posterior Cingulate	Limbic	3.07	125
	Precuneus	Parietal	3.04	306
	Cuneus	Occipital	2.71	206
	Mid Temporal Gyrus	Temporal	2.53	150
	Angular Gyrus	Parietal	2.34	32
	Cingulate Gyrus	Limbic	2.34	35
	Sup Temporal Gyrus	Temporal	2.29	108
	Lingual Gyrus	Occipital	2.11	84
	Supramarginal Gyrus	Parietal	2.1	28

iCAP	Region	Lobe	Z-value	voxels
	Paracentral Lobule	Frontal	1.88	36
	Sup Par Lobule	Parietal	1.86	43
	Putamen	Sub-lobar	1.85	10
	Sup Frontal Gyrus	Frontal	1.82	74
**18**	Sup Par Lobule	Parietal	2.9	85
	Postcentral Gyrus	Parietal	2.76	149
	Precuneus	Parietal	2.6	94
	Sup Frontal Gyrus	Frontal	2.55	275
	Paracentral Lobule	Frontal	2.49	42
	Mid Frontal Gyrus	Frontal	2.49	193
	Caudate Caudate Body	Sub-lobar	2.45	34
	Inf Par Lobule	Parietal	2.43	78

Table 1 – continued from previous page

iCAP	Region	Lobe	Z-value	voxels
	Sup Occipital Gyrus	Occipital	2.01	22
	Mid Occipital Gyrus	Occipital	1.94	39
	Inf Par Lobule	Parietal	1.92	36
	Inf Temporal Gyrus	Occipital	1.74	10
	Med Frontal Gyrus	Frontal	1.73	15
	Cingulate Gyrus	Limbic	2.57	161
	Sup Frontal Gyrus	Frontal	2.39	199
	Mid Frontal Gyrus	Frontal	2.3	325
	Med Frontal Gyrus	Frontal	2.3	91
	Putamen	Sub-lobar	2.14	33
11	Sup Temporal Gyrus	Temporal	2.13	84

iCAP	Region	Lobe	Z-value	voxels
	Precentral Gyrus	Frontal	2.39	100
	Parahippocampal Gyrus	Limbic	2.39	13
	Med Frontal Gyrus	Frontal	2.27	59
	Thalamus	Sub-lobar	2.27	33
	Cingulate Gyrus	Limbic	2.18	51
	Cuneus	Occipital	2.01	38
	Inf Frontal Gyrus	Frontal	1.96	12
	Paracentral Lobule	Parietal	2.92	57
	Precuneus	Parietal	2.65	406
	Lingual Gyrus	Occipital	2.53	28
	Sup Par Lobule	Parietal	2.53	125

19

Table 1 – continued from previous page

iCAP	Region	Lobe	Z-value	voxels	iCAP	Region	Lobe	Z-value	voxels
	Mid Temporal Gyrus	Temporal	2.11	74		Angular Gyrus	Parietal	2.49	22
	Mid Occipital Gyrus	Occipital	2.04	50		Mid Temporal Gyrus	Temporal	2.39	166
	Precentral Gyrus	Frontal	2.04	81		Cingulate Gyrus	Limbic	2.31	48
	Inf Temporal Gyrus	Occipital	2.03	21		Fusiform Gyrus	Temporal	2.31	31
	Insula	Sub-lobar	2.02	35		Postcentral Gyrus	Parietal	2.28	79
	Fusiform Gyrus	Temporal	1.99	17		Inf Temporal Gyrus	Occipital	2.24	21
	Inf Par Lobule	Parietal	1.96	88		Supramarginal Gyrus	Parietal	2.2	12
	Inf Frontal Gyrus	Frontal	1.96	110		Inf Par Lobule	Parietal	2.19	136
	Precuneus	Parietal	1.95	100		Cuneus	Occipital	2.08	95
	Postcentral Gyrus	Parietal	1.93	60		Mid Occipital Gyrus	Occipital	1.98	38
	Caudate	Sub-lobar	1.93	25		Sup Temporal Gyrus	Temporal	1.97	46
	Cuneus	Occipital	1.89	28		Posterior Cingulate	Limbic	1.93	10

Table 1 – continued from previous page

iCAP	Region	Lobe	Z-value	voxels	iCAP	Region	Lobe	Z-value	voxels
	Sup Par Lobule	Parietal	1.87	18		Sup Frontal Gyrus	Frontal	1.88	38
	Supramarginal Gyrus	Parietal	1.83	11		Precentral Gyrus	Frontal	1.84	14
	Paracentral Lobule	Frontal	1.81	21		Mid Frontal Gyrus	Frontal	1.8	58
	Thalamus	Sub-lobar	1.75	17	20	Postcentral Gyrus	Parietal	4.96	82
	Ant Cingulate	Limbic	1.75	10		Sup Par Lobule	Parietal	4.43	82
12	Sup Occipital Gyrus	Occipital	2.75	24		Precentral Gyrus	Frontal	3.52	47
	Fusiform Gyrus	Occipital	2.59	60		Precuneus	Parietal	2.78	96
	Mid Occipital Gyrus	Occipital	2.53	155		Sup Frontal Gyrus	Frontal	2.69	158
	Inf Temporal Gyrus	Temporal	2.45	51		Mid Frontal Gyrus	Frontal	2.43	134
	Cuneus	Occipital	2.43	164		Med Frontal Gyrus	Frontal	2.1	15
	Mid Temporal Gyrus	Temporal	2.2	234		Inf Par Lobule	Parietal	2.06	14
	Caudate	Sub-lobar	2.19	7		Cuneus	Occipital	1.94	28

Table 1 – continued from previous page

iCAP	Region	Lobe	Z-value	voxels	iCAP	Region	Lobe	Z-value	voxels

Table 1: We compute the average z-score and the total number of voxels occupied in brain areas defined with Talairach Client.

Supplementary Method

Participants. Fourteen healthy volunteers participated in the study. Data acquisitions were obtained with a Siemens 3T Trio TIM scanner, using a 32-channel head coil. The structural images were acquired using a high resolution three-dimensional T1-weighted MPRAGE sequence (160 slices, TR/TE/FA = 2.4 s/2.98 ms/9°, matrix = 256 x 240, voxel size = 1 x 1 x 1.2mm^3). For the resting-state fMRI data, subjects were instructed to lie still and relax in the scanner with their eyes are closed. The total acquisition took around 8 minutes. The data were acquired using gradient-echo echo-planar imaging (TR/TE/FA = 1.1s/27ms/90°, matrix = 64 x 64, voxel size = 3.75 x 3.75 x 5.63mm^3, 21 slices, 450 volumes). The first 10 volumes are discarded in order to assure the magnetization stability.

FMRI Data Processing. FMRI data is preprocessed using in-house MATLAB code combined with SPM8 (FIL,UCL,UK) and IBASPM toolboxes [1]. First, fMRI volumes were realigned to the first scan and spatially smoothed with Gaussian filter (FWHM=5mm). We used further motion correction to mark the time points with high frame-wise displacement [2], and performed cubic spline interpolation around these time points. We did not remove those frames since TA algorithm exploits the continuity of fMRI time courses to deconvolve the effect of the hemodynamic response. Finally, we excluded two subjects with high motion, therefore, the results were obtained from twelve healthy controls in total. The anatomical images are coregistered onto the functional mean image and segmented (NewSegment, SPM8) for the six different MNI templates. The anatomical automatic labeling (AAL) atlas [3], composed of 90 regions without the cerebellum, was mapped onto each subject's coregistered anatomical image and further downsampled to match the native space of the functional images. The anatomical atlas is only used to guide the spatial regularization of the Total Activation framework.

Total Activation. The TA framework [4] allows obtaining denoised and well-behaving reconstructions of the activity-related, activity-inducing, and innovation signals from noisy fMRI measurements by using state-of-the-art regularization that takes the L_1-norm of the activity-related signal after applying a well-chosen differential operator. The differential operator Δ_L includes the inverse operator of the hemodynamic system together with a first-order derivative. The inverse hemodynamic operator is adapted from the formulation in [5] based on the first-order Volterra-series

approximation of non-linear Balloon model [6,7]. TA also combines the temporal with spatial regularization; i.e., we impose mixed-norm constraint to promote (but not enforce) coherent activations inside anatomically defined regions whereas sparse activations across regions.

In essence, TA reverts to convex optimization that combines least-square data fitting with the two regularization terms:

$$\tilde{\mathbf{x}} = \arg\min_{\mathbf{x}} \frac{1}{2} \|\mathbf{y} - \mathbf{x}\|_F^2 + \mathcal{R}_T(\mathbf{x}) + \mathcal{R}_S(\mathbf{x}), \tag{1}$$

where

$$\mathcal{R}_T(\mathbf{x}) = \sum_{i=1}^{V} \lambda_1[i] \left\| \frac{\Delta_L\{\mathbf{x}[i,\cdot]\}}{\sqrt{\sum_{t=1}^{N} |\Delta_L\{x[i,t]\}|}} \right\|_1, \tag{2}$$

and

$$\mathcal{R}_S(\mathbf{x}) = \sum_{t=1}^{N} \lambda_2[t] \left\| \frac{\Delta_{\text{Lap}}\{\mathbf{x}[\cdot,t]\}}{\sqrt{\sum_{k=1}^{M} \sqrt{\sum_{i \in R_k} \Delta_{\text{Lap}}\{x[i,t]\}^2}}} \right\|_{(2,1)}, \tag{3}$$

where Δ_{Lap} is the spatial Laplacian operator, $\lambda_{1,2}$ are the regularization parameters, $\|\mathbf{x}\|_F$ is the Frobenius norm and Δ_{Lap} is the Laplacian filter. We use generalized forward-backward splitting [8], for denoising case also known as parallel Dykstra-like proximal algorithm [9], to solve the optimization problem in (1). The joint solution is obtained by incorporating the proximal maps of both spatial and temporal regularizations [4,10].

TA is applied to every subject in his own native functional space, and the results are subsequently normalized (using SPM8) to a common MNI space as to be able to determine group results.

Surrogate data analysis. For each subject, we generated a surrogate dataset by phase randomizing every voxel time course, and then apply TA with exactly the same settings. In Supplementary Fig. S1, we depict the histograms of the surrogate data and the original data. The histograms of phase-randomised BOLD signal and the original BOLD signal are very similar whereas theys change considerably after TA regularization, which is indicative of the non-random nature of block-type activity in the real data. We plot the log-plot of innovations in order to highlight the difference between the two histogram profiles to accommodate for the large number of data points.

Temporal clustering of transients. There is no baseline in the innovation signals, that is, every activation is relative with respect to the previous time point. We first separate the innovation signals between activation and de-activation, which are represented by the positive and negative peaks, respectively. Then, we flipped the sign of negatives and concatenated these time points to perform a temporal k-means clustering. In order to determine the time points of interest, we followed a two-step procedure (for space and time) based on the surrogate data that accounts for the subject effect. Specifically, for each subject, we generated a surrogate data by phase randomization of the original BOLD signals of each voxel and run TA on the surrogate data. Then, again for each subject we selected two thresholds as 99% and 1% from the histogram of innovations signals of each subjects surrogate data. Similar to [11], after thresholding, we kept the voxels that are connected with 26 neighborhood and 6 voxels. Finally, we sum the ‘active’ time points at each time point and only selected the time points where there were at least 500 active voxels in space (that threshold corresponds to around 4% of the whole volume) [12]. In Supplementary Fig. S2 a, we show the first step of thresholding; that is, for each voxel’s time course, the time points that exceed the threshold (determined by surrogate dataset) are marked as ‘active’. Supplementary Fig. S2 b and c illustrate the global thresholding (here shown for one subject) of the positive and negative transients, respectively. The average number of volumes included in k-means clustering constitute around 28% of the all time points (1521 positives, 1477 negatives, total 2998 (56%) out of 5280 scans).

We then performed k-means clustering using cosine distance as the similarity criteria. The cosine distance between two vectors can be regarded as the non-normalised version of correlation where the mean is kept, as

$$C_d(\mathbf{x}, \mathbf{y}) = \frac{\langle \mathbf{x}, \mathbf{y} \rangle}{\|\mathbf{x}\|_2 \|\mathbf{y}\|_2}. \tag{4}$$

Here, the reasons of using cosine distance are twofolds: 1) it is well adapted for non-negative signals, and 2) it does not modify the baseline. Finally, we empirically set the number of clusters to 20 by using a leave-one-subject-out cross-validation scheme (see iCAP activation maps in Supplementary Fig. S3–S4 and the cost function in Supplementary Fig. S9). Inspection of the iCAPs’ spatial and temporal patterns, we deduced that the first 13 clusters were particularly stable across subjects and the last 7 clusters had less consistency.

The iCAPs’ maps were computed by combining and averaging the clusters of positive and negative (sign

flipped) transients. Since the distribution of the maps are not symmetric, in order to be able to compute z-scores, we subtracted the mode (the maximum value of the histogram) instead of the mean and divide by the standard deviation. The time course of each cluster was computed by back-projecting the iCAPs onto the sustained activity-inducing signals. The back-projection was computed separately for positive and negative weights in order to minimize the effect of spatial linear dependency.

Amount of spatial overlap through Jaccard Distance. The similarity measure was computed via Jaccard distance, which is the total intersection between two binary patterns normalised by each individual pattern; i.e., ratio of intersection to union.

$$J_d(i, j) = \frac{\#\{\text{iCAP}_i = 1\} \cap \{\text{iCAP}_j = 1\}}{\#\{\text{iCAP}_i = 1\} \cup \{\text{iCAP}_j = 1\}}.$$

Binarized maps are obtained by thresholding according to z-score $\geq$1.5. Significant intersections are determined as follows: we generate surrogate data by spatially permuting the binary maps, where instead of voxel-wise permutation we exploited a block-permutation to keep the spatial structure (blocks of size $6 \times 10 \times 5$ voxels). Then, we performed a non-parametric test using the maximum statistics and computed the significance p$\leq$0.01. Supplementary Fig. S5 shows the spatial overlap matrix; stars indicate statistically significant connections.

Spatial correlates of traditional DMN We computed the conventional DMN using seed-region based correlation with the seed in PCC; MNI coordinates (0,-53,26), averaged over a $7 \times 7 \times 11\text{mm}^3$ neighborhood. The conventional DMN was derived for each subject and then averaged to obtain the group-level conventional DMN. We computed the spatial similarity between the iCAPs and group-level conventional DMN using cosine distance. The subject-level similarities are shown in Supplementary Fig. 8.

Temporal overlap. Total and average durations of iCAPs were computed from the normalized iCAPs time courses (absolute $|z| \geq 1$, see Supplementary Fig. S8 for the histograms). We counted the number of 'active' iCAPs at each time point regardless of the sign of the weight, and observed the distribution over number of iCAPs versus total time. We then specified which iCAP combinations occur mostly for different number of overlapping iCAPs (Fig. 5

and Supplementary Fig. 9 for weight dependent combinations and histogram at level of overlap). For overlapping iCAPs (i.e., from two to five), the iCAP distribution and the most frequent 20 iCAP combinations are illustrated with their respective activated (red) and deactivated (blue) state. For instance, the most frequent combination at level two is the DMN (positive) and anterior salience network (negative), followed by DMN (negative) and secondary visual (positive).

References

1. Alemán-Gómez, Y., Melie-Garćia, L. & Valdés-Hernandez, P. IBASPM: Toolbox for automatic parcellation of brain structures. *12th Annual Meeting of the Organization for Human Brain Mapping* **27** (2006).

2. Power, J. D., Barnes, K. A., Snyder, A. Z., Schlaggar, B. L. & Petersen, S. E. Spurious but systematic correlations in functional connectivity MRI networks arise from subject motion. *NeuroImage* **59**, 2142 – 2154 (2012).

3. Tzourio-Mazoyer, N. *et al.* Automated anatomical labeling of activations in SPM using a macroscopic anatomical parcellation of the MNI MRI single-subject brain. *NeuroImage* **15**, 273–289 (2002).

4. Karahanoglu, F. I., Caballero-Gaudes, C., Lazeyras, F. & Van De Ville, D. Total activation: fMRI deconvolution through spatio-temporal regularization. *NeuroImage* **73**, 121 – 134 (2013).

5. Khalidov, I., Fadili, J., Lazeyras, F., Van De Ville, D. & Unser, M. Activelets: Wavelets for sparse representation of hemodynamic responses. *Signal Processing* **91**, 2810–2821 (2011).

6. Friston, K. J., Mechelli, A., Turner, R. & Price, C. J. Nonlinear responses in fMRI: The balloon model, Volterra kernels, and other hemodynamics. *NeuroImage* **12**, 466 – 477 (2000).

7. Buxton, R. B., Wong, E. C. & Frank, L. R. Dynamics of blood flow and oxygenation changes during brain activation: The Balloon model. *Magnetic Resonance in Medicine* **39**, 855–864 (1998).

8. Raguet, H., Fadili, J. & Peyre, G. A generalized forward-backward splitting. *SIAM Journal on Imaging Sciences* **6**, 1199–1226 (2013).

9. Combettes, P. Iterative construction of the resolvent of a sum of maximal operators. *Convex Analysis* **16**, 727–748 (2009).

10. Karahanoglu, F. I., Bayram, I. & Van De Ville, D. A signal processing approach to generalized 1-D total variation. *IEEE Transactions on Signal Processing* **59**, 5265 –5274 (2011).

11. Liu, X. & Duyn, J. H. Time-varying functional network information extracted from brief instances of spontaneous brain activity. *Proceedings of the National Academy of Sciences* **110**, 4392–4397 (2013).

12. Petridou, N., Gaudes, C. C., Dryden, I. L., Francis, S. T. & Gowland, P. A. Periods of rest in fMRI contain individual spontaneous events which are related to slowly fluctuating spontaneous activity. *Human Brain Mapping* **34**, 1319–1329 (2013).
